# Bile diversion to the distal small intestine has comparable metabolic benefits to bariatric surgery

**DOI:** 10.1038/ncomms8715

**Published:** 2015-07-21

**Authors:** Charles Robb Flynn, Vance L. Albaugh, Steven Cai, Joyce Cheung-Flynn, Phillip E. Williams, Robert M. Brucker, Seth R. Bordenstein, Yan Guo, David H. Wasserman, Naji N. Abumrad

**Affiliations:** 1 Department of Surgery, Vanderbilt University Medical Center, 1161 21st Avenue South, MCN CC2308, Nashville, Tennessee 37232-2730, USA.; 2 Rosalind Franklin University, North Chicago, Illinois 60064, USA.; 3 Department of Biological Sciences, Vanderbilt University, Nashville, Tennessee 37235-1634, USA.; 4 Department of Pathology, Microbiology, and Immunology, Vanderbilt University, Nashville, Tennessee 37232-2561, USA.; 5 Center for Quantitative Sciences, Department of Cancer Biology, Vanderbilt University, Nashville, Tennessee 37232-6848, USA.; 6 Department of Molecular Physiology & Biophysics, Vanderbilt University Medical Center, Nashville, Tennessee 37232-6303, USA.

## Abstract

Roux-en-Y gastric bypass (RYGB) is highly effective in reversing obesity and associated diabetes. Recent observations in humans suggest a contributing role of increased circulating bile acids in mediating such effects. Here we use a diet-induced obesity (DIO) mouse model and compare metabolic remission when bile flow is diverted through a gallbladder anastomosis to jejunum, ileum or duodenum (sham control). We find that only bile diversion to the ileum results in physiologic changes similar to RYGB, including sustained improvements in weight, glucose tolerance and hepatic steatosis despite differential effects on hepatic gene expression. Circulating free fatty acids and triglycerides decrease while bile acids increase, particularly conjugated tauro-β-muricholic acid, an FXR antagonist. Activity of the hepatic FXR/FGF15 signalling axis is reduced and associated with altered gut microbiota. Thus bile diversion, independent of surgical rearrangement of the gastrointestinal tract, imparts significant weight loss accompanied by improved glucose and lipid homeostasis that are hallmarks of RYGB.

## Abstract (web summary)

Gastric bypass surgery is one of the most effective interventions to achieve durable weight loss. Here, Flynn *et al*. show that, in mice, bile diversion to the small intestine results in beneficial and sustained metabolic improvements similar to Roux-en-Y gastric bypass surgery.

## Graphical abstract

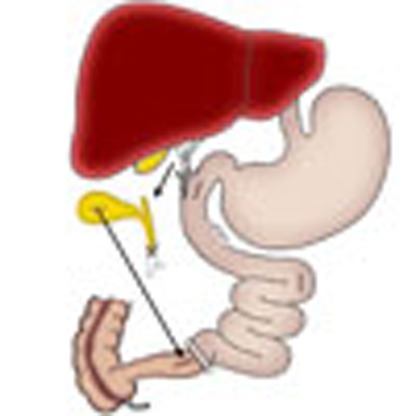

With obesity approaching epidemic proportions, bariatric surgery has undergone steady growth worldwide. Procedures such as the biliopancreatic diversion (BPD) and duodenal switch operations have significant weight loss and diabetes resolution; however, this is at the cost of severe malabsorption and nutritional complications that effectively limits their use. On the other hand, bariatric procedures such as Roux-en-Y gastric bypass (RYGB) and vertical sleeve gastrectomy (VSG) are the most commonly used in the United States with RYGB being the more effective for durable treatment of severe obesity and type 2 diabetes^1^,^2^. The RYGB, which connects a 30 ml stomach pouch to the mid-jejunum, bypassing the entire foregut, imparts both weight loss-dependent and -independent benefits. Several studies have examined the mechanisms involved in early and late improvements following RYGB and VSG. These include, but are not limited to, caloric restriction, rapid delivery of nutrients to the distal intestine, bypass of foregut peptide secretion, altered enterohepatic circulation of bile acids and weight loss^3^,^4^,^5^,^6^,^7^,^8^. In humans, an increase in circulating bile acids is observed as early as 10 days after bariatric surgery and strongly correlates with improved glucose tolerance and lipid metabolism^7^,^8^,^9^,^10^. Both RYGB and VSG increase circulating bile acid levels, suggesting their involvement in the metabolic benefits obtained with these procedures^6^,^10^,^11^. This is supported by studies in rodents in which interposition of a segment of distal ileum within the proximal jejunum improves insulin resistance while increasing circulating primary and secondary bile acids^12^,^13^. Similarly, catheter-mediated bile diversion to the mid-distal jejunum in the rat induces weight loss, decreases adiposity, improves glucose tolerance and increases circulating bile acids^14^.

Bile acids are produced by the liver, stored in the gallbladder (GB) and secreted into the duodenum where they act as amphipathic emulsifiers to facilitate absorption of dietary fats. In addition to this digestive role, bile acids are now known to function as signalling molecules to regulate their own synthesis, energy metabolism, glycemic control and immunity^15^,^16^,^17^,^18^,^19^,^20^. The effects of bile acids on gene transcription are mediated by the farnesoid X receptor (FXR) that is abundant in the ileum and liver^21^, where it serves to downregulate hepatic lipogenesis and bile acid synthesis and export. Seeley and co-workers^22^ recently reported in diet-induced obese (DIO) mice that the full effects of VSG on weight loss and glucose tolerance were diminished with FXR deficiency.

Bile acids strongly interact with the gut microbiota and this interaction is closely linked to metabolic status. In the large intestine, bacteria modify bile acids to yield secondary compounds such as the alpha and beta muricholic acid (α- and β-MCA), which antagonize FXR^23^. Microbiota regulation of FXR occurs primarily in the ileum and proximal colon. Fibroblast growth factor 15 (FGF15; FGF19 in humans) downstream of FXR mediates the consequent effects on liver bile acid metabolism^23^,^24^. Conversely, diet-induced alterations in the bile acid pool influence gut microbiota composition and activity in bile acid modification^25^. Alterations in the microbiota consequent to dietary factors are thought to be potential contributors to the pathogenesis of obesity and type 2 diabetes^26^,^27^,^28^. High fat diet (HFD) in humans^29^ and rodents^27^ increases the ratio of Firmicutes/Bacteroidetes, two major intestinal bacterial populations. The reduction in Bacteroidetes, species active in bile acid deconjugation would contribute to less diversity of the bile acid pool and possibly to negative metabolic effects. When faecal microbiota from human twins discordant for obesity was transplanted into germ-free mice, they resulted in reproducible transmission of the donor metabolic phenotypes^30^. A few studies in rodents and humans demonstrated significant alterations in gut microbiota following RYGB^31^,^32^,^33^,^34^,^35^ and suggested that they significantly contribute to the benefits realized after the procedure; whether altered bile acid metabolism is a factor underlying these changes was not explored.

Although the evidence accumulated to date strongly supports metabolic benefits of bile acid signalling, it is unclear if they can be used effectively for treating DIO and co-morbidities such as diabetes. In particular, and mainly reflecting the difficulty in establishing rodent models of RYGB and bile diversion, it remains unresolved if diverting bile flow to enhance bile acid signalling in the ileum can realize benefits that are as dramatic and sustained as those observed with bariatric surgery. Here we used a mouse model of high fat DIO and comprehensively examined side-by-side over an 8-week period the impact of RYGB compared with diverting bile acids to either the jejunum or ileum on body weight, adiposity, glucose homeostasis and liver metabolism. The effect of the bile acid diversion site on the gut microbiota profile was also examined. We show that when compared in this manner, bile diversion to the ileum results in metabolic remission that is equal or slightly superior to RYGB. The changes reflect a combination of decreased fat absorption, altered enterohepatic bile acid signalling and reversal of the gut microbiota to a lean-like profile.

## Results

### Biliary diversion to the illeum and RYGB reduce body weight

**Figure 1:**
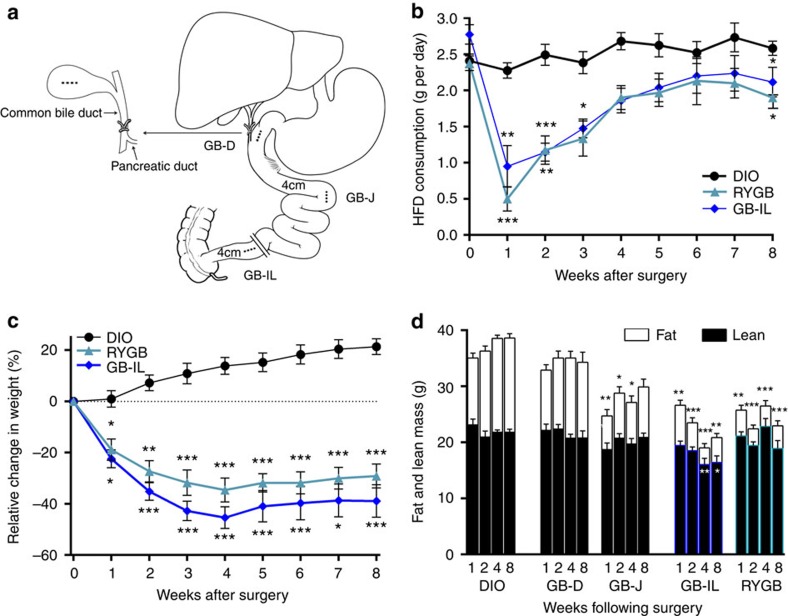
Biliary diversion schematic and effects on body weight, food intake and body composition. (**a**) For the biliary diversion procedure, the common bile duct was ligated proximal to the pancreatic duct. The gallbladder was then anastomosed to one of the following: (1) jejunum 4 cm distal to the ligament of Treitz (GB-J), (2) ileum 4 cm proximal to the ileo-caecal valve (GB-IL) or (3) gallbladder-duodenal anastomosis (GB-D model) at the level of the ampulla of Vater. GB-D was performed without significant alteration of bile flow and functioned as a sham surgery. The RYGB procedure was performed as we previously described^31^. Mice were fed a high fat diet (HFD) for induction of diet-induced obesity (DIO), underwent the surgical procedures and were monitored for 8 weeks post-operatively. (**b**) Average daily food intake, Bio-Serv F3282 (5.49 kcal g^−1^); (**c**) relative change in body weight in *N* of 15 DIO, 15 GB-IL and 7 RYGB mice; and (**d**) serial body composition measures via NMR of fat and lean mass (*N* of 15 DIO, 12 GB-D, 11 GB-J, 15 GB-IL, 12 RYGB). Values shown are mean±s.e.m. *P<0.05, **P<0.01, ***P<0.001 versus DIO controls by one-way analysis of variance with Dunn's post-test.

To determine the effects of bile diversion on weight loss and reversal of metabolic dysregulation, we developed three mouse models in which the GB was anastomosed to the duodenum (GB-D), jejunum (GB-J) or ileum (GB-IL) (Fig. 1a). The GB-D served as the sham control, as these mice have a surgical anastomosis without physiologic bile diversion. Three additional groups were studied; an RYGB group^36^, a DIO control and control mice pair-fed to the GB-IL cohort.

The relative efficacy of biliary diversion or RYGB on food intake (Fig. 1b), body weight (Fig. 1c) and body composition (Fig. 1d) was assessed in a mouse model of HFD-induced obesity (DIO). At 1 week post-operative, all mice that underwent surgical procedures including GB-IL and RYGB mice had decreased food consumption (Fig. 1b), consistent with the post-operative period. At 2 weeks the GB-D and GB-J mice increased their food consumption indicating surgical recovery; however, food intake in GB-IL and RYGB mice remained significantly less (Supplementary Fig. 1a). Beyond 2 weeks, mice subjected to the GB-D and GB-J procedures displayed increasing body weight reaching DIO levels by 8 weeks; however, GB-IL and RYGB mice had sustained decreases in food intake (Fig. 1b and Supplementary Fig. 1a). Surprisingly, mice receiving GB-IL procedures exhibited weight loss equal to or greater than that observed with RYGB throughout the remainder of the study. Consistent with the changes in body weight, mice in the GB-IL and RYGB groups had significant reductions in total body fat mass (Fig. 1d). Pair-feeding to the GB-IL cohort did not lead to weight loss similar to the GB-IL group (Supplementary Fig. 1b), collectively suggesting that GB-IL and RYGB induce weight loss through mechanisms beyond reductions in food intake alone.

### Biliary diversion to the ileum increases serum bile acids

**Figure 2:**
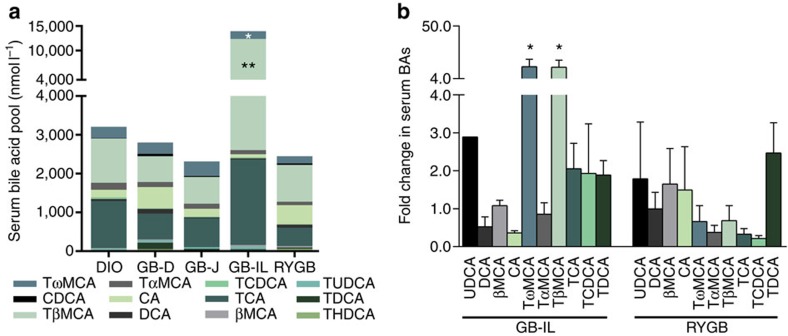
Biliary diversion modifies bile acid abundance and composition. (**a**) Serum bile acid levels and (**b**) fold change in serum bile acids in mice subjected to biliary diversion (GB-D, GB-J and GB-IL) and RYGB relative to DIO controls at 8 weeks post-operative. *P<0.05, **P<0.01 versus DIO by one-way analysis of variance with post-test. Values shown are mean±s.e.m. *N* of 5 per group.

To examine the potential effects of bile acid diversion on circulating bile acid levels, we measured total and fractionated bile acid concentrations in DIO controls and bile diverted mice 8 weeks post-operative. Serum bile acid levels were not significantly different among GB-D, GB-J or RYGB cohorts compared with DIO mice (Fig. 2a, Supplementary Figs 2–3 and Supplementary Tables 1–2). However, GB-IL mice showed nearly 10-fold increase in total bile acid levels that were driven primarily by increased tauro-ω-MCA (TωMCA) and tauro-β-MCA (TβMCA). CA content did not significantly differ among the groups. Relative to DIO controls, serum concentrations of most other taurine-conjugated bile acids tended to be higher in GB-IL but the increases were not significant (Fig. 2b). Surprisingly, we did not detect significant increases in any individual bile acid or total bile acid species after RYGB. Thus, biliary diversion to the ileum specifically increased the conjugated bile acid species, TωMCA and TβMCA—the latter being a potent FXR antagonist^23^,^37^.

### Biliary diversion to the ileum improves metabolism

**Figure 3:**
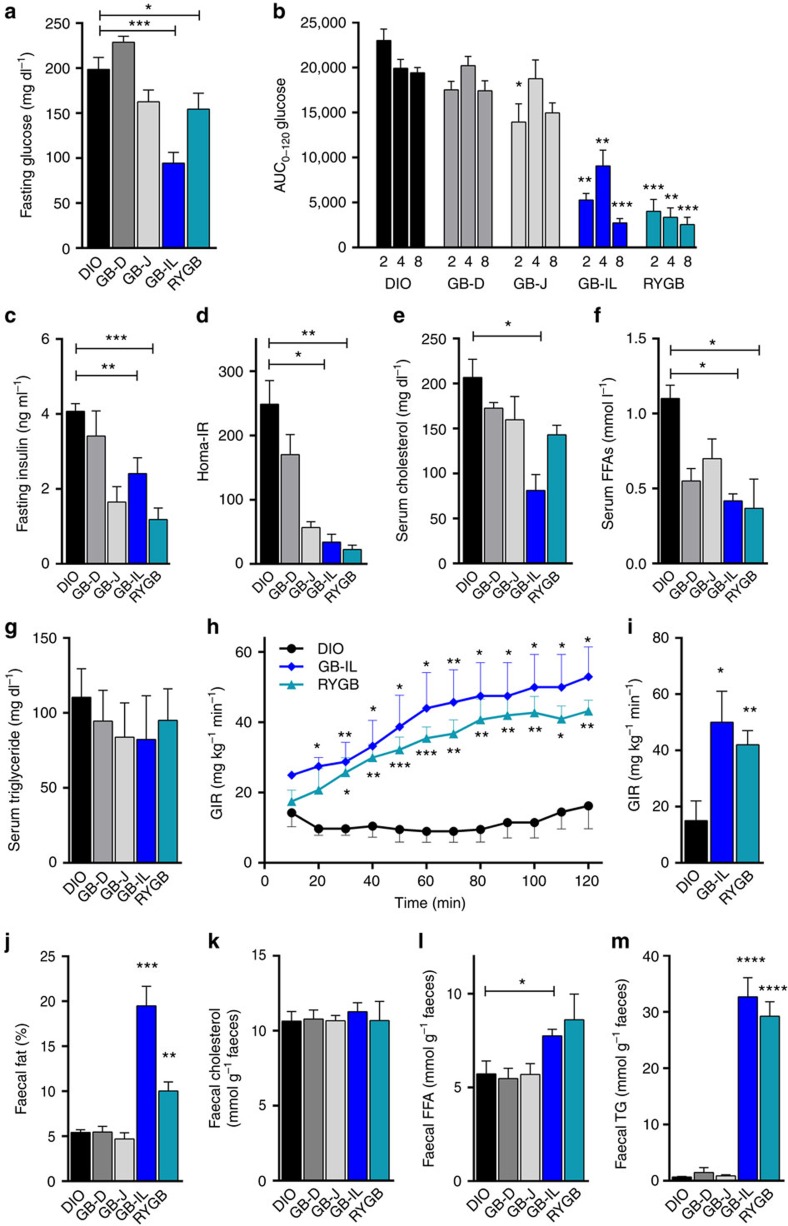
Effects of biliary diversion on glucose tolerance, insulin sensitivity and lipid metabolism. (**a**) Blood glucose was measured after a 4-h period of food-restriction. (**b**) Experimental groups underwent intraperitoneal glucose challenge at 2, 4 and 8 weeks post-operative following biliary diversion procedures. Area under the curve (AUC) measurements between 0 and 120 min were calculated and compared among groups. (**c**) Fasting plasma insulin, (**d**) the homeostatic model assessment of insulin resistance (HOMA-IR) (**e**) serum cholesterol, (**f**) serum FFAs, (**g**) serum triglycerides determined at 4 weeks post-operative with GB-J, GB-IL and RYGB compared with DIO. (**h**,**i**) Insulin sensitivity was determined by the hyperinsulinemic-euglycemic clamp, where a continuous infusion of insulin (4 mU kg^−1^ min^−1^) was delivered with euglycemia (140 mg dl^−1^) maintained by a variably adjusted glucose infusion. (**h**) Glucose infusion rates (mg glucose per kg min^−1^) over the 120 min procedure in DIO, GB-IL and RYGB mice (*N*=4), (**i**) and the mean glucose infusion rates during the last 20 min of the clamp. (**j**) Faecal fat (w/w% by mass), (**k**) faecal cholesterol (mol per g faeces); (**l**) faecal FFA (mol per g faeces) and (**m**) faecal triglyceride (mol per g faeces) in mice under study. *P<0.05; **P<0.01; ***P<0.001; ****P<0.0001 versus DIO by one-way analysis of variance with Dunn's post-test. Values shown are mean±s.e.m. (for **a**,**b**, *N* of 15 DIO, 17 GB-D, 18 GB-J, 23 GB-IL, 6 RYGB; for **c**,**d; j**–**m**, *N* of 5–10 per group).

To explore the role of biliary diversion on glucose homeostasis, glucose tolerance tests were performed in all groups (Fig. 3). Fasting blood glucose levels measured at 4 weeks post-operatively were significantly lower in GB-IL and RYGB mice as compared with GB-D and GB-J mice (Fig. 3a). Blood glucose excursions following intraperitoneal glucose challenge at 2, 4 and 8 weeks post-operatively were all significantly lower with GB-IL and RYGB mice as compared with GB-D, GB-J or DIO controls, indicative of improved glucose tolerance. While the GB-J procedure elicited improved glucose tolerance at 2 weeks, the improvements were not sustained by 4 weeks post-operatively (Fig. 3b, Supplementary Fig. 4). Fasting plasma insulin (Fig. 3c) was decreased in the GB-IL group, approaching levels observed in RYGB mice. The changes in glucose and insulin levels corresponded to significantly decreased HOMA-IR, suggesting improved insulin sensitivity (Fig. 3d). Other circulating fasting metabolites also showed differences relative to DIO, with serum cholesterol being decreased in GB-IL but not RYGB, and serum free fatty acids (FFAs) significantly decreased in both GB-IL and RYGB (Fig. 3e,f). Fasting triglycerides did not differ among groups (Fig. 3g). To better define the potential changes in insulin sensitivity, we subjected separate cohorts of DIO, RYGB and GB-IL mice to hyperinsulinemic-euglycemic clamps. Consistent with improvements in whole-body insulin sensitivity, glucose infusion rates over time (Fig. 3h) and averaged during the steady state phase of the clamp (Fig. 3i) were significantly increased in RYGB and GB-IL mice compared with DIO. Thus, the effects of RYGB and GB-IL on whole-body glucose metabolism are a reflection of improvements in insulin action.

The observed decreased circulating FFAs may be reflective of either improved insulin sensitivity or incomplete absorption of fats from the gut. To assess for changes in dietary fat absorption, we collected faeces from these mice and extracted total lipid content, as well as measured faecal cholesterol, FFAs and triglycerides (Fig. 3j–m). GB-IL and RYGB had significant increases in total faecal lipids compared with DIO (Fig. 3j), which corresponded to significant increases in faecal FFAs (Fig. 3l) and triglycerides (Fig. 3m) without a change in faecal cholesterol (Fig. 3k). Overall, the data confirm that there is significant fat malabsorption in both the RYGB and GB-IL models.

### Biliary diversion to the ileum increases energy expenditure

**Figure 4:**
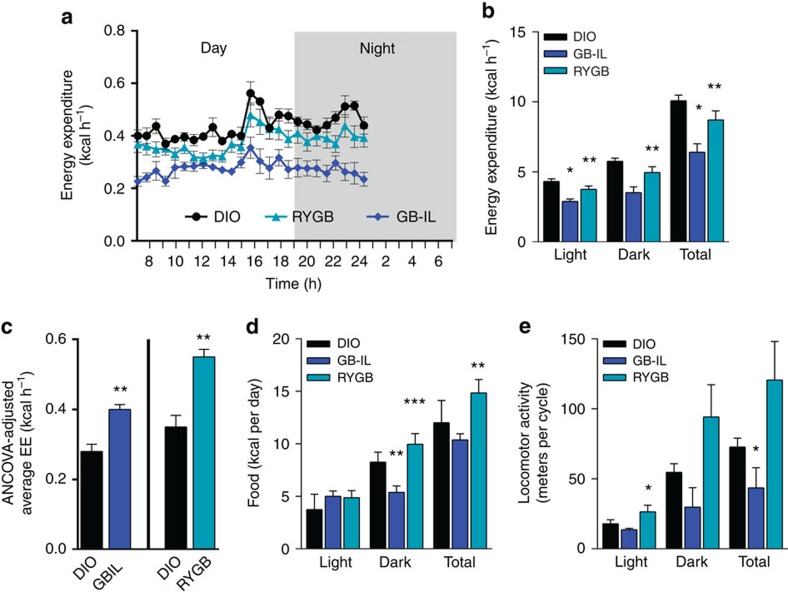
Energy expenditure in response to bariatric procedures. (**a**) Energy expenditure over a 24-h period was assessed by indirect calorimeter in DIO, GB-IL and DIO mice at 4 weeks post-operative. (**b**) Unadjusted energy expenditure (kcal h^−1^). (**c**) ANCOVA adjusted mean energy expenditure. (**d**) Food intake (kcal per day) was monitored daily for 5 days in each study group. (**e**) The frequency of locomotor activity (pedestrian meters) as determined by beam breaks per 24-h period. *N* of 4 DIO, *N* of 6 GB-IL, *N* of 6 RYGB per group. Values shown are the mean±s.e.m. *P<0.05 by two-way ANCOVA.

Bile acids are significantly elevated post bariatric surgery in humans^6^,^7^,^8^,^9^,^38^,^39^ and in our GB-IL model (Fig. 2) and have been shown to increase energy expenditure (EE) through TGR5-mediated processes in skeletal muscle and adipose tissue^40^,^41^,^42^. To explore whether changes in EE also contributed to the metabolic improvements in this model, we subjected cohorts of DIO, RYGB and GB-IL mice to 24 h indirect calorimetry under standard conditions at 4 weeks post-operative (Fig. 4a). Analysis of covariance (ANCOVA) was used to adjust EE variables for the group differences in body weight. Measurements of activity, lean mass, fat mass, food intake and locomotor activity were also made concurrently. At the time of calorimetry, RYGB and GB-IL groups weighed significantly less than DIO controls (26.6±1.1 g for RYGB; 26.2±2.4 g for GB-IL and 45.3±2.3 g for DIO; mean±s.e.m., *N*=4). On a per mouse basis, EE (kcal h^−1^) in GB-IL mice (0.29±0.057) and RYGB mice (0.39±0.064) was less than DIO controls (0.45±0.031) (Fig. 4b). ANCOVA was used to assess the impact of bariatric procedures after adjusting for total body mass (TBM) or fat-free mass (FFM) (Supplementary Fig. 5, Supplementary Table 3). Both GB-IL and RYGB mice, relative to DIO controls, displayed increased, ANCOVA model-predicted EEs values (Fig. 4c). Daily food intake (Fig. 4d) was significantly lower during the dark cycle in GB-IL, but was significantly higher in RYGB in both the dark cycle and during the total 24 h period. The frequency of locomoter activity showed a similar differential trend with mice receiving GB-IL displaying a lower number of pedestrian metres travelled compared with DIO or RYGB (Fig. 4e). Locomotor activity in RYGB mice tended to be greater than that in DIO controls.

### Effects of surgical procedures on hepatic gene expression

**Figure 5:**
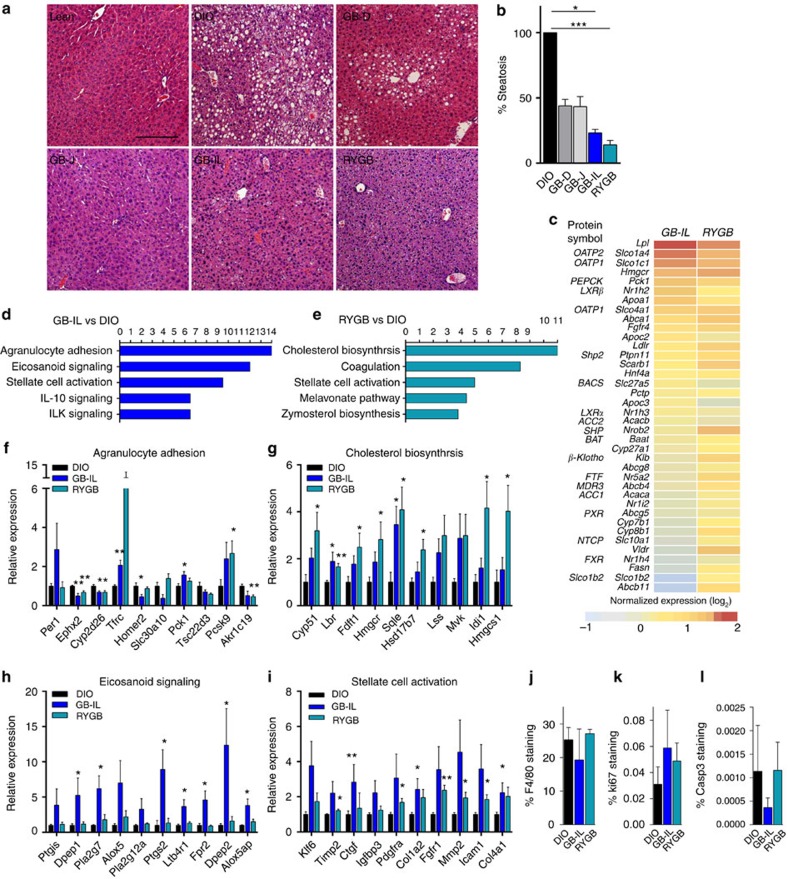
Effects of biliary diversion on liver histology and hepatic gene expression. (**a**) Liver steatosis as assessed by H&E staining in lean, DIO, GB-D, GB-J, GB-IL and RYGB mice at 8 weeks after surgery was significantly reduced (**b**) in GB-IL and RYGB mice relative to DIO. (**c**) Expression of FXR target genes in liver at 4 weeks post-operative. (**d**,**e**) Canonical pathways most differentially expressed between GB-IL versus DIO (**d**) and RYGB versus DIO (**e**). Expression of hepatic genes involved in (**f**) agranulocyte adhesion, (**g**) cholesterol biosynthesis, (**h**) eicosanoid signalling and (**i**) stellate cell activation. (**j**) Inflammation by hepatic F4/80, (**k**) Ki-67 and (**l**) caspase 3 by immunohistochemical staining. *P<0.05; **P<0.01, ***P<0.001 versus DIO by one-way analysis of variance with Dunn's post-test. Values are mean±s.e.m. (*N* of 5–10 per group for **b**–**i**; 4 per group for **j**–**l**). Magnification bar, 200 μm.

Hepatic steatosis is a frequent comorbidity of obesity and high fat feeding. Livers were harvested from all mice groups 8 weeks post-operatively for histologic analyses (Fig. 5a). The comparison included age- and gender-matched high fat or chow fed controls. As expected, HFD resulted in severe hepatic steatosis compared with the chow control diet. GB-J or GB-D mice displayed significant reductions in hepatic steatosis relative to DIO mice. However, more marked reductions in hepatic steatosis were observed after GB-IL and RYGB (Fig. 5b).

To more closely examine the hepatic changes associated with by the whole-body metabolic improvements and the apparent hepatic resistance to steatosis, we examined changes in hepatic gene expression by RNA-seq (Fig. 5c–i; Supplementary Fig. 6). Genes potentially regulated by bile acids were manually interrogated for expression changes with GB-IL or RYGB (Fig. 5c). Similar to a recent report^43^, lipoprotein lipase (*Lpl*) was most significantly upregulated (increased 2.4-fold relative) in mice treated with a gut-restricted FXR agonist, fexaramine, relative to DIO controls. The bile acid transporters, *Slco1a4* (Oatp2) and *Slco1c1* (Oatp1) were similarly upregulated in both GB-IL and RYGB groups. Such increases were in the context of relatively unchanged *FXR* expression. An unbiased interrogation of the mRNA transcriptome (Ingenuity Pathway Analysis, Qiagen) was conducted (Fig. 5d–i). The top three canonical pathways most increased in GB-IL versus DIO livers (Fig. 5d) included agranulocyte adhesion (Fig. 5f), eicosanoid signalling (Fig. 5h) and stellate cell activation (Fig. 5i). Those pathways that changed the greatest in RYGB versus DIO included cholesterol biosynthesis (Fig. 5g), coagulation and stellate cell activation (Fig. 5i). Some markers of NF-κB were significantly increased in GB-IL and/or RYGB mice (Supplementary Fig. 7a). Interleukin-1b was only increased in GB-IL. Lipogenic targets were marginally affected in either GB-IL or RYGB (Supplementary Fig. 7b). Several apoptotic and inflammatory genes also trended upward in GB-IL more so than RYGB (Supplementary Fig. 7c,d). To determine whether such gene expression changes translated into phenotypic differences, we also surveyed by immunohistochemistry markers of inflammation by F4/80 (Fig. 5j; Supplementary Fig. 8d–f), proliferation by Ki-67 (Fig. 5k; Supplementary Fig. 8g–i) and apoptosis by caspase 3 staining (Fig. 5l, Supplementary Fig. 8j–l). The significant individual gene expression profiles did not appear to translate into effects on histologic phenotypes. Overall the data suggest that both RYGB and GB-IL do not result in substantially improved inflammatory markers as compared with DIO despite the complete reversal of hepatic steatosis; however, there were trends toward increased apoptotic and inflammatory responses.

### Effects of surgical procedures of BA transporters

**Figure 6:**
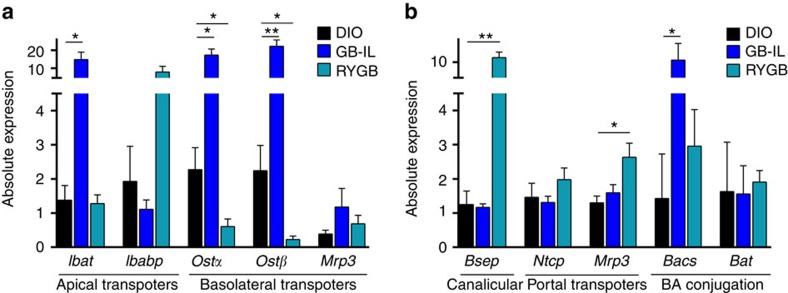
Effects of biliary diversion on ileal and hepatic gene expression. (**a**) Ileum and (**b**) liver tissues 8 weeks post-operative were harvested for gene expression analysis using RT–PCR. *P<0.05, **P<0.01 versus DIO by one-way analysis of variance followed by two-tailed Students *t*-test. Values shown are mean±s.e.m. *N* of 3–5 per group. *Bacs*, bile-acid-CoA synthetase; *Bat*, bile acid transporter; *Bsep*, bile sale effluent pump; *Ibabp*, ileal bile acid-binding protein; *Ibat*, Ileal bile acid transporter; *Mrp3*, multi-drug resistance protein 3; *Ntcp*, Na-taurocholate cotransporting polypeptide; *Ostα* and *Ostβ*, organic solute transporter α and β.

To gain insight into the mechanisms underlying the metabolic adaptations observed after GB-IL and RYGB mice as compared with DIO controls, we measured expression of bile acid transporters in the ileum and liver with additional measurements of conjugation enzymes in the liver. In the ileum (Fig. 6a), GB-IL but not RYGB markedly increased the apical ileal bile acid transporter (*Ibat*, up 10-fold) and the basolateral bile acid transporters *Ostα* and *Ostβ* (organic solute transporter α and β). GB-IL only increased the apical bile acid-binding protein. In contrast, in the liver, RYGB but not GB-IL strongly increased expression of the canalicular bile salt export pump and the portal multi-specific organic ion transporter 3 (Fig. 6b). Thus, the effect of GB-IL is predominantly on ileal bile acid transport, while RYGB enhances expression of liver bile acid transporters. RYGB, and to a greater extent GB-IL, tended to increase hepatic expression of bile-acid-CoA synthetase, the enzyme facilitating conjugation of CA with taurine, but these increases were not significant. These data are consistent with the enhanced circulation of bile acids with GB-IL.

### Altered hepatic FXR/FGF15 signalling

**Figure 7:**
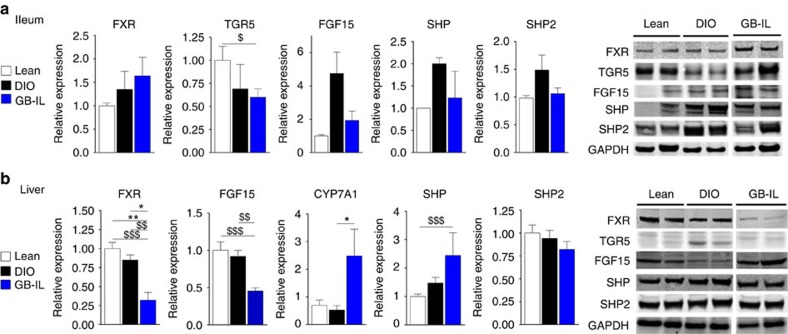
Signalling in the ileum and liver is altered after biliary diversion. Immunoblots of (**a**) ileum and (**b**) liver protein obtained from lean, DIO and GB-IL mice 8 weeks after surgery. Expression was normalized to GAPDH. *P<0.05, **P<0.01 by one-way analysis of variance with Dunn's post-test. ^$^*P*<0.05, ^$$^*P*<0.01, ^$$$^*P*<0.001 by two-tailed Students *t*-test. Values shown are mean±s.e.m. *N* of 3–8 per group.

Given that GB-IL procedure alone was associated with increased circulating bile acids and with increased mRNA expression of bile acid transporters (Fig. 6), we next examined the effect of GB-IL compared with lean and DIO controls with respect to bile acid signalling proteins in the ileum and liver (Fig. 7). FXR expression in the ileum trended upward after GB-IL, while that of TGR5 trended downward (Fig. 7a). GB-IL mice displayed reduced levels of FGF15, a postprandial hormone induced by bile acid and released from the small intestine into the portal circulation where it acts to repress hepatic bile acid synthesis. Expression of the small heterodimer partner (SHP) and the non-receptor tyrosine phosphatase (Shp2) in ileums of GB-IL mice was restored towards levels observed in lean chow fed mice.

It is well established that ileal reabsorption and enterohepatic circulation of bile acids negatively regulate hepatic bile acid synthesis by repressing expression of the rate-limiting enzyme cholesterol 7α-hydroxylase 1 (Cyp7a1)^42^. This effect is likely mediated by increased levels of FGF15 that bind to FGF receptor 4, leading to transactivation of SHP and Shp2 (refs 44, 45). Alternatively, bile acids, acting through FXR, can directly inhibit hepatic Cyp7a1 expression. Thus, under conditions of absent or low bile acids, Cyp7a1 drives bile acid synthesis via the classical pathway to restore bile acid levels; thus, high bile acid availability should decrease the expression of Cyp7a1 leading to repressed bile acid synthesis^46^.

Eight weeks after surgery, GB-IL displayed significantly reduced levels of hepatic FXR relative to lean and DIO controls (Fig. 7b). Consistent with the suppression of FGF15, hepatic expression of Cyp7a1 was dramatically increased in GB-IL mice. The downstream FXR target SHP was slightly increased while the differential expression of Shp2 was not observed across the groups. This pattern of protein expression and the associated decreased FGF15 would be expected with low bile acid availability, in contrast with the increased circulating bile acids we observed with GB-IL (Fig. 2).

### Adaptations of the gut microbiome to bariatric procedures

**Figure 8:**
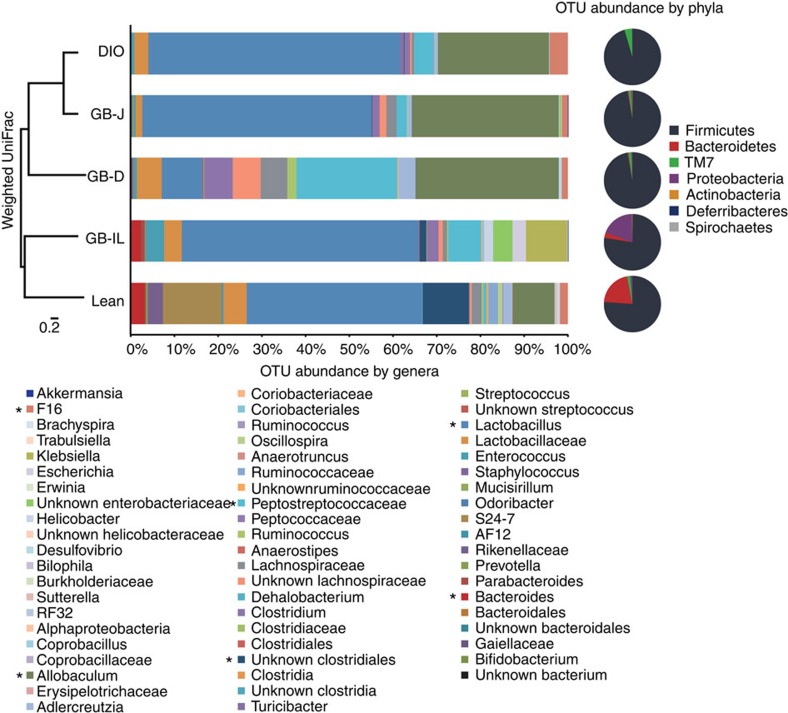
Biliary diversion procedures differentially alter the gut microbiome. Caecal contents from DIO, GB-D, GB-J and GB-IL mice 8 weeks after surgery were subjected to 16S rRNA gene sequence analysis. Relative abundance of bacterial genera (bar chart) and phyla (pie chart) with each surgical procedure is shown. The *y*-axis is a weighted Unifrac analysis of the microbiota for the pooled treatment group. ‘*' indicates reference points for a given taxa (*N* of 5 mice per group).

The microbiota influence bile acid metabolism and excretion^47^, which raises the question whether the change in microbiota is causing the altered bile acid pool in GB-IL. We examined if significant changes in the microbiota occur with bile diversion and if they might explain some of the observed changes. Caecal contents from mice 8 weeks after the biliary diversion procedures were subjected to pyrosequencing, targeting the V3–V4 region of the 16S ribosomal RNA (rRNA) gene^48^. Figure 8 illustrates the breakdown of the bacterial taxonomy at the genera and phyla level. The dominant phylum, Firmicutes, represented 95.5–97.1% of the sequences observed in the DIO, GB-D and GB-J mice, while they only represented 76.0–77.3% of the sequences for the lean and GB-IL mice. The DIO, GB-D and GB-J mice were nearly void of Bacteroidetes, consistent with previous reports that Bacteroidetes bacteria are lower in obese mice and people than in lean controls^31^. In contrast, GB-IL mice had a significant increase in Bacteroidetes thus decreasing the ratio of Firmicutes/Bacteroidetes in a manner similar to that described for bariatric procedures^49^. DIO, GB-D and GB-J mice had marked increases in the relative abundance of the TM7 phyla that was not present in GB-IL mice. GB-IL also had a significant increase in Proteobacteria relative to other groups. Pairwise analysis of the weighted UniFrac (sensitive to the abundances of taxa) indicates that there was a significant difference between the microbiota of GB-IL and the GB-D or GB-J mice (Supplementary Table 4). Each surgical procedure resulted in similar levels of microbial diversity (Supplementary Fig. 9a,b and Supplementary Table 5) but lean mice had the most diverse microbiome overall (*P*<0.05). The individual mice tended to cluster within the treatment groups, with regard to their weighted beta diversity (Supplementary Fig. 9c). To further investigate more specific bacterial strains that might be at work, we probed by 16 rRNA gene reverse transcription (RT–PCR) the abundance of *Christensenella minuta* in DIO, bile diverted and RYGB mice caecal DNA. This leptogenic bacterial species, recently identified as being strongly associated with the lean phenotype in humans^50^, was not detectable in any caecal DNA sample in contrast to *C. minuta* genomic DNA positive controls, therefore these findings relegate this species as minimally relevant to the metabolic improvements observed in this model.

## Discussion

An important role of bile acid signalling in regulating energy metabolism and improving insulin resistance is well supported by recent evidence^51^,^52^,^53^. In this study using a biliary diversion mouse model, we examined the metabolic benefits of bile acids as a function of the intestinal site of bile diversion. The changes in food intake, fat absorption, bile acid signalling and gut microbiota remodelling in the bile diversion mice were compared with those obtained with RYGB, the most common bariatric procedure associated with sustained reversal of diabetes^54^. Among the biliary procedures, diversion of bile flow to the ileum (GB-IL) resulted in the most significant improvements in glucose and lipid metabolism and these were sustained over the 8-week duration of the study. The benefits included weight loss, fat mass reduction, improved glucose tolerance, improved insulin sensitivity and resolution of liver steatosis. These improvements were comparable to those observed after RYGB. However, in contrast to RYGB, bile diversion to the ileum elicited changes in total EE that were independent of TBM. Thus, bile diversion to the ileum confers metabolic benefits similar to RYGB without compromising intestinal continuity. In our hands, bile acid levels after RYGB were not significantly increased.

Bile acid diversion to the ileum (GB-IL) achieves metabolic improvements that are superior to those observed with more proximal intestinal diversions (for example, GB-J) and even, by some metrics, to those after RYGB. GB-IL, as compared with GB-J, resulted in ∼30% less food intake, 40% lower body weight and a body composition similar to that of lean controls. In addition, although the GB-IL mice were maintained on a HFD, glucose tolerance and hepatic lipid content were preserved at levels identical to those measured in lean controls. Pair-feeding did not reconstitute these same improvements, indicating that mechanisms besides hypophagia are at work in this model. This result is consistent with the previous finding that diverting bile to the jejunum improved adiposity and insulin resistance better than a pair-fed sham control^11^,^14^.

Bile acids secreted into the duodenum act as emulsifiers that facilitate absorption of dietary fat by increasing formation of intestinal micelles, aiding the function of pancreatic lipases. It is clear from our studies that a significant, *albeit* partial, component of the metabolic improvements with biliary diversion (Fig. 3), is related to fat malabsorption, evidenced by the increased levels of total faecal fat in GB-IL and the decreased circulating plasma cholesterol and FFA levels. As one would expect fat absorption in the absence of bile is retarded, likely because the pancreatic lipase does not work efficiently in this environment. From a clinical perspective, this impairment of fat absorption is somewhat similar to the BPD procedure popularized by Scopinaro *et al*.^55^. Those patients have significant malabsorption and nutritional deficiencies, presumably because both the biliary and pancreatic secretions are diverted so far distally in the small bowel. In our procedure, only the biliary secretions are diverted to the distal small bowel with the pancreatic secretions maintaining contact with the gastrointestinal tract. The animals tolerated the procedure and did not become anaemic over time.

Bile acid diversion, particularly GB-IL, was associated with enhanced bile acid reabsorption as evident by the marked increases in serum bile acids and by the expression of ileal bile acid transporters Ibats, *Ostα* and *Ostaβ* (Fig. 6a). These findings contrast with those observed with RYGB, where plasma bile acids and ileal bile acid transporters were not increased. The increased availability of circulating bile acids in the GB-IL group and consequent activation of the nuclear bile acid receptor, FXR, would be expected to increase ileal production of FGF15, which migrates to the liver and facilitates inhibition of the rate-limiting enzyme Cyp7a1 to reduce bile acid synthesis via the classical pathway^46^. However, while trending greater relative to DIO, the enhanced expression of FXR in GB-IL mice was not significant. We also did not detect in GB-IL compared with DIO an increase in ileal FGF15 protein content. Instead, hepatic expression of FXR was downregulated resulting in upregulation of Cyp7a1 (Fig. 7a). Taken together these changes are suggestive of other regulatory non-classical pathways involved in bile acid homeostasis. Chen *et al*.^56^ identified an upstream modifier, Shp2, coordinating bile acid stimulation of FXR. The lack of increase in hepatic expression of Shp2 associated with a drop in hepatic expression of FXR would have been expected to result in decreased expression of the SHP; instead we observed significantly increased SHP expression (Fig. 7b), suggesting the existence of additional non-classical alternative pathways involved in bile acid synthesis. This mechanism could be related to the large increases in circulating TβMCA levels observed with GB-IL antagonizing hepatic FXR and leading to relief of FXR-mediated suppression of bile acid synthesis as was previously reported^23^,^57^

There are a number of mechanisms in the GB-IL mouse model that appear to be contributing to the improved metabolic phenotype. Both cohorts, GB-IL and RYGB, compared with DIO, showed significant increases in EE when adjusted for total body weight (Fig. 4e). Unlike RYGB, however, the changes in EE after GB-IL were independent of TBM. These increased metabolic rates after both GB-IL and RYGB were concurrent with significant reductions in food intake in the presence of malabsorption of dietary lipids (Fig. 1b) and locomotor activity (Fig. 4g). GB-IL was also associated with changes in the expression of genes regulating hepatic inflammation although such changes were not readily evident histologically. Taken together, these results indicate that bile diversion to the ileum, unlike RYGB, induces a distinct metabolic phenotype characterized by increased circulating bile acids and a body-weight-independent control of EE that is consistent with the described peripheral TGR5-mediated pathway of homeostatic control^42^. It is possible that excessive bile acid signalling via TGR5 could have been the early driver of these events in the immediate post-operative period, while being later downregulated by other homeostatic mechanisms that aim to preserve body weight. Alternatively, the improved metabolic profile might be related to the effects of the markedly increased bile acids on the gut microbiota.

An increasing body of evidence supports a role of the imbalanced gut microbiota that is induced by HFDs in the development of obesity and its comorbidities^27^,^29^,^58^. We found that high fat feeding decreased microbial diversity and the proportion of Bacteroidetes, Actinobacteria and Proteobacteria, while it increased that of Firmicutes. The observed increase in the ratio of Firmicutes/Bacteroidetes after weight loss is consistent with previous reports. Interestingly, high fat feeding resulted in increased presence of the pro-inflammatory TM7 bacteria, previously reported to be increased in type 2 diabetes^59^,^60^. These high fat induced changes in the microbiota were partially reversed after GB-IL as the mice retained some Bacteroidetes while also expanding their Proteobacteria content^61^. In addition, the GB-IL mice lost the pro-inflammatory phyla TM7 species, which was retained in all the other groups. The changes in our study were not associated with altered *C. minuta*, recently associated with the lean phenotype in human^50^. However, the combined normalization of lipid and glucose homeostasis after bile diversion implicates stabilization of a leptogenic gut microbial community and inhibition of ‘inflammatory' enterobacteria through a bile acid-mediated mechanism reminiscent of the effects elicited by TβMCA after administration of the antioxidant tempol to obese mice^37^. A recent report on remodelling of the obese intestinal microbiome with the antioxidant tempol showed similar microbiome changes that were correlated with increased TβMCA levels^37^.

Overall, our findings in rodents indicate that the biliary diversion model is as effective as RYGB in reversing most negative complications of DIO and provides a useful tool for dissecting the complex mechanisms that lead to weight loss and diabetes resolution. Translation of our findings from rodents to humans will need much additional research related to the long-term effects of the procedure. For example, despite resolution of hepatic steatosis, we observed increases in hepatic expression of genes related to eicosanoid production (GB-IL, Fig. 5h) and stellate activation (both RYGB and GB-IL, Fig. 5i) and potential long-term effects of these changes on liver function will require further investigation.

From a clinical standpoint, while biliary diversion to the ileum is a procedure that is less technically demanding than RYGB, the need to interrupt the bile flow through the common bile duct impacts significant risk to the reversibility of such a procedure with at least a choledochojejunostomy or hepaticojejunostomy. Moreover, the diversion procedure as presented here is not possible in the many diabetic and/or obese individuals who have previously had a cholecystectomy for gallstone disease. At this time, although our findings suggest the potential usefulness of applying the biliary diversion procedure to humans this needs to await additional data that are not currently available on its long-term safety, efficacy and tolerability.

In conclusion, biliary diversion to the distal small intestine results in sustained improvements in body weight, adiposity, liver steatosis, as well as glucose and lipid metabolism. Although some improvements are observed with bile diversion to the proximal intestine, they are not stable and less significant. Multiple mechanisms appear to account for the benefits of bile diversion to the ileum and they include hypophagia, reduced dietary lipid absorption, increased total bile acid pool consequent to more ileal bile acid reabsorption combined with enhanced bile acid synthesis and maintenance of a more lean-like microbiome. This model gives novel insight into the mechanisms potentially at work in RYGB clinically. These findings require further investigation and warrants careful examination of the therapeutic potential of bile diversion in obese humans.

## Methods

### Mice and operations

All experiments and surgical preparations were performed according to protocols approved by the Vanderbilt University Medical Center Institutional Animal Care and Use Committee (IACUC). The mice remained under the care of the Division of Animal Care (DAC) at Vanderbilt University in compliance with NIH guidelines and the Principles of Laboratory Animal Care, and the Guide for the Care and Use of Laboratory Animals. Male C57BL/6 mice were housed at 23 °C on a 0700–1900-hour light cycle and were fed a HFD (60% kcal from fat; Bio-Serv, Frenchtown, NJ), starting at 6 weeks of age for 12 weeks prior to being randomly allocated to a surgical group. Biliary diversion was performed under isoflurane anaesthesia using a 12-15 × microsurgical scope. In the biliary diversion operation, a side-to-side, running-continuous, GB to small bowel anastomosis was created on the anti-mesenteric surface of the small bowel using 9-0 nylon suture. The anastomosis was created on the undersurface of the GB, away from the fundus and from the delicate vasculature supplying the GB. The length of the anastomosis was ∼2–3 mm. Bile flow was diverted from the GB to either the duodenum (via a GB-D at the level of the ampulla of Vater, labelled) or the jejunum (via a GB-J, 4 cm distal to the ligament of Treitz) or the ileum (via GB-IL, 4 cm proximal to the ileo-caecal valve). The GB-D model was nearly identical to the natural physiologic bile flow and does not change pancreatic flow; hence the GB-D is considered the sham procedure. RYGB was performed as we previously reported^31^. The common bile duct was ligated using 9-0 nylon as far proximal to the pancreatic duct as possible as not to alter pancreatic secretory flow. Abdominal fascia and skin were closed using simple, interrupted sutures of 5-0 polyglactin and 6-0 polypropylene sutures, respectively. Mice were maintained on the HFD and water *ad libitum* after surgery up to the endpoint measurements. Early surgical mortality (<1 week post-operatively) was ∼15%, being almost entirely due to leakage of the GB-to-bowel anastomosis regardless of its location. In earlier studies this rate was higher (∼50%), though as the surgeons became more experienced with the GB-to-bowel anastomosis (>6–9 months of experience) this improved greatly. Surgical success rate, defined as mouse survival for >1 week without surgical complications, was 80% for GB-D, 85% for GB-J, 90% for GB-IL and 75% for RYGB. Surgical complications included obstruction at the site of the GB anastomosis to the jejunum or ileum (<5%) and occurred within 1–4 weeks post surgery. In our experience, 40% of mice subjected to RYGB mice and 80% of those that undergo BPD have a reduced haematocrit^31^. GB-intestinal anastomosis was not associated with anaemia, as we previously reported for RYGB^36^.

### Whole-body tissue composition

Body mass was measured weekly for 8 weeks using mq10 NMR analyzer (Bruker Optics Inc., Billerica, MA) following 2 h of fasting^31^. Fat and muscle mass were calculated in grams.

### Intraperitoneal glucose tolerance tests (IPGTT)

Mice were fasted for 4 h prior to IPGTT at 2, 4 or 8 weeks after surgery. Blood was sampled from the tail vein before and 10, 20, 30, 45, 60, 75, 90 and 120 min after an intraperitoneal injection of dextrose (20%) at 2.0 mg g^−1^ body weight. Blood glucose levels (mg dl^−1^) were measured using a blood glucose meter (SureStep, LifeScan, Inc.).

### Indirect calorimetry

We assessed EE by indirect calorimetry using a system comprised of 16 identical, yet separate, metabolic cages equipped for the continual monitoring of ambulatory activity and *ad libitum* access to HFD and water (Promethion, Sable Systems, Las Vegas, NV). Oxygen (O_2_), carbon dioxide (CO_2_) and water vapour levels were constantly monitored while temperature and humidity levels were tightly regulated (GA3, Sable Systems)^61^. The incurrent air flow rate was set at 3000, ml min^−1^ (FR8, Sable Systems). CO_2_ consumption and O_2_ production were measured for each mouse at 10-min intervals for 1 min. Respiratory quotients were calculated as the ratio of CO_2_ production over O_2_ consumption. EE is calculated using the Weir equation (1):


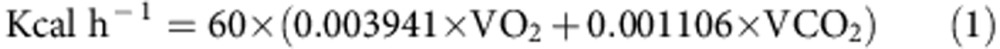


Data acquisition and instrument control were coordinated by MetaScreen v. 2.2.18 and the obtained raw data was processed using ExpeData v. 1.7.30 (Sable Systems) using an analysis script detailing all aspects of data transformation^62^ . The script is available on request from the corresponding author. Data was analysed by ANCOVA using scripts available at the National Mouse Metabolic Phenotyping Centers (MMPC, Nashville, TN, USA) Energy Expenditure Analysis Page (www.mmpc.org/shared/regression.aspx; accessed March 2015). Such methods consider EE differences when adjusting for differences in body mass/composition.

### Chemicals

CA (5β-cholanic acid-3α,7α,12α-triol), α-MCA (5β-cholanic acid-3α,6β,7α-triol), β-MCA (5β-cholanic acid-3α,6β,7β-triol), chenodeoxycholic acid (5β-cholanic acid-3α,7α-diol, CDCA), deoxycholic acid (5β-cholanic acid-3α,12α-diol, DCA), hyodeoxycholic acid (5β-cholanic acid-3α,6α-diol, HDCA), ursodeoxycholic acid (5β-cholanic acid-3α, 7β-diol, UDCA), taurocholic acid (5β-cholanic acid-3α,7α,12α-triol-N-[2-sulphoethyl]-amide, TCA), tauro-α-MCA (5β-cholanic acid-3α, 6β,7α-triol-N-[2-sulphoethyl]-amide), TβMCA (5β-cholanic acid-3α,6β,7β-triol-N-[2-sulphoethyl]-amide), TωMCA (5β-cholanic acid-3α,6α,7β-triol-N-[2-sulphoethyl]-amide), taurochenodeoxycholic acid (5β-cholanic acid-3α,7α-diol-N-[2-sulphoethyl]-amide, TCDCA), taurodeoxycholic acid (5β-cholanic acid-3α, 12α-diol-N-[2-sulphoethyl]-amide, TDCA), taurohyodeoxycholic acid (5β-cholanic acid-3α,6α-diol-N-[2-sulphoethyl]-amide], THDCA), and taurolithocholic acid (5β-cholanic acid-3α-ol-N-[2-sulphoethyl]-amide, TLCA) were purchased from Steraloids, Inc. (Newport, RI). Cholic-2,2,4,4-d4 acid (5β-cholanic acid-3α,7α,12α-triol-2,2,4,4-d4, d4-CA), taurocholic-2,2,4,4-d4 acid (5β-cholanic acid-3α,7α,12α-triol-N-[2-sulphoethyl]-amide, TCA-d4), chenodeoxycholic-2,2,4,4-d4 acid (5β-cholanic acid-3α,7α-diol-2,2,4,4-d4, CDCA-d4); glycocholic-2,2,4,4-d4, (5β-cholanic acid-3α,7α,12α-triol-N-[carboxymethyl]-amide-2,2,4,4-d4, GCA-d4), glycochenodeoxycholic-2,2,4,4-d4 acid (5β-cholanic acid-3α,7α-diol-N- [carboxymethyl]-amide-2,2,4,4-d4, GCDCA-d4) were purchased from C.D.N. Isotopes Inc. (Pointe Claire, Montreal, PQ, CA). TβMCA (5β-cholanic acid-3α,6β,7β-triol-N-[2-sulphoethyl]-amide-2,2,4,4-d4, TβMCA-d4), was purchased from United States Biological Corp., Swampscott, MA). HPLC grade water, acetonitrile, ethanol, methanol, ammonium acetate and ammonia were purchased from Sigma Chemicals (St. Louis, MO). Formic acid was purchased from Thermo Scientific (Rockford, IL).

### Calibrators and controls

Stock solutions of 2.5 mmol l^−1^ of all bile acids (THCA, HCA, TαMCA, TβMCA, TωMCA, HDCA, THDCA: 10 mmol l^−1^) were used to prepare calibrators with concentrations of 100 μmol l^−1^ in methanol. For the preparation of calibrators, bile acids were mixed to achieve final concentrations of 20, 2.5, 0.75, 0.25, 0.05, 0.015 and 0.005 μmol l^−1^. To prepare 20 ml of a 2.0 nmol l^−1^ internal standard, 250 μl each of d4-CDCA, d4-TCA, d4-GCDCA and 500 μl d4-CA, d4-TβMCA and d4-GCA were added to 20% (v/v) acetonitrile.

### Sample preparation for UPLC/ESI-MS

To 50 μl of plasma were added 200 μl of 100 mM aqueous sodium hydroxide and 50 μl of internal standard. The sample was heated at 64 °C for 30 min, centrifuged for 10 min at 14,400*g* and the supernatant acidified to pH 7.0 with 50 μl of 0.1 M hydrochloric acid. The sample was brought to a final volume of 1 ml with water and applied to a 1cc (30 mg) Oasis HLB cartridge (Waters, Milford, MA) previously equilibrated first with 1 ml of methanol, then 1 ml of water. The column-bound bile acids were washed with 1 ml of 5% (v/v) aqueous methanol then 1 ml of 2% (v/v) aqueous formic acid. Bile acids were eluted from the column with 1 ml of 2% (v/v) ammonia in methanol and the eluent evaporated to dryness using a rotary evaporator at 30 °C for 2 h. Samples were resuspended in 100 μl of 25% (v/v) acetonitrile in water.

### Liquid chromatography

An Acquity ultra performance liquid chromatography system (UPLC; Waters) employing a Luna C18(2) 50 × 2.0 mm, 3 mm column, C18 4 × 2.0 mm pre-column, both from Phenomenex (Torrance, CA), was heated to 50 °C, and a binary solvent system of 20% (v/v) acetonitrile in water (mobile phase A) and 80% (v/v) acetonitrile in water (mobile phase B) both containing 1 mM ammonium acetate were used to resolve plasma bile acids. The injection volume onto the column was 15 μl. The flow rate was 400 μl min^−1^ into the mass spectrometry (MS). Chromatography was similar to a published method and started with a solvent mixture of 95% A that decreased to 85% A at 15 min, to 75% at 20 min, then to 25% at 22 min where after it increased to 95% A at 24 min for 3 min^63^.

### Mass spectrometry

MS analysis was performed using a TSQ Quantum mass spectrometer (ThermoFinnigan, Sunnydale, CA) equipped with an electrospray ionization (ESI) probe in negative-ion mode. Quantitation was done in a multiple reaction monitoring mode with collision energy of 10 V. The following (optimized) parameters were used for the detection of the analytes and the internal standard; N2 sheath gas, 49 p.s.i.; N2 auxiliary gas, 25 p.s.i.; spray voltage, 3.0 kV; source CID, 25 V; capillary temperature, 300 °C; capillary offset, −35 V; tube lens voltage, 160 V; Q2 gas pressure, 1.5 mtor; Q3 scan width 1 *m/z*; Q1/Q3 peak widths at half-maximum, 0.7 *m/z*. Calibration curves and concentration of individual bile acids were calculated by LCQuan 2.5.5 software (ThermoFinnigan). Concentrations of individual bile acids were calculated from peak area in the chromatogram detected with SRM relative to the appropriate internal standard. The composition and amount of bile acids in serum are reported in Supplementary Tables 1 and 2 and Supplementary Figs 6 and 7.

### Liver tissue macroarray assembly and immunohistochemistry

For assessments of steatosis, Image J was used to quantify the amount of steatosis in 5–6 liver images for each surgical group and control. Tissue microarray (TMA) construction and biomarker staining was performed by the Vanderbilt Translational Pathology Shared Resource. One millimetre cores were sampled, in duplicate, from each of 70 liver specimens donor blocks using TMA Grand Master Tissue Microarrayer (Perkin Elmer, Waltham, MA) and randomly arranged on the TMA. Liver micrograph images were captured using a high throughput Leica SCN400 Slide Scanner automated digital image system from Leica Microsystems (Buffalo Grove, IL). Whole slides were imaged at × 20 magnification to a resolution of 0.5 μm per pixel. Tissue cores were mapped using Ariol Review software. The numbers of positive (brown) and negative (blue) nuclei were determined by analysis of the high-resolution images in the Ariol software. Immunostained TMA slides were imaged on a Leica SCN400 Slide Scanner (Leica Biosystems). Tissue cores were imaged at × 20 magnification to a resolution of 0.5 μm per pixel. Cells were identified utilizing standard Ariol analysis scripts (Leica). Upper and lower thresholds for colour, saturation, intensity, size, roundness and axis length were set for both blue haematoxylin staining of nuclei and for brown DAB reaction products (anti-mouse F4/80, ab6640, Abcam, Cambridge, MA). Thus, brown (diaminobenzidine) positive cells can be distinguished from blue (haematoxylin only) negative cells. The area of positive staining per core was calculated as a per cent of the total analysed area divided by area of brown (DAB positive) pixels.

### RNA sequencing, data processing and analysis

The transcriptional sequencing was implemented with modifications of the standard Illumina methods^64^. Sequencing reactions were performed using the Illumina HiSeq (v3 chemistry). Initial QC quantification of the extracted total RNA was done by using Qubit Fluorometer (Invitrogen). Agilent 2100 Bioanalyzer was used to check the quality of the extracted RNA from both the samples. About 30 ng of total RNA was amplified using the NuGEN Technologies Ovation RNA amplification kit optimized for RNA sequencing. The output is double-stranded complementary DNA (cDNA) that was sheared using the Covaris instrument to an average insert size of 300 bp. The sheared DNA entered the standard Illumina TruSeq library preparation protocol at the standard end-repair step. Following end polishing, a single ‘A'-base was added to the 3′-end of the DNA fragments. This prepared the DNA fragments for ligation to specialized adaptors that have a ‘T'-base overhang at their 3′-ends. The Illumina protocol relies on the ligation of the TruSeq adaptor sequences to each end of the DNA molecules to facilitate ligation-mediated PCR (LM-PCR) amplification of the resulting material. The end-repaired DNA with a single ‘A'-base overhang was ligated to the adaptors in a standard ligation reaction using T4 DNA ligase and 2–4 uM final adaptor concentration, depending on the DNA yield following purification after the addition of the ‘A'-base (a 10-fold molar excess of adaptors was used in each reaction). Following ligation, the samples were purified and amplified with 12 cycles of LM-PCR to amplify the ligated material in preparation for cluster generation. Following LM-PCR, the samples were purified and assessed for quality using the Agilent Bioanalyzer. Final quantitation was performed using the KAPA Biosystems Kapa Library Quant kit. Based on the RT–PCR data, final 10 nM library dilutions were generated and sequenced at a cluster density of 950,000 clusters per mm^2^.

Multiple stages of quality control (QC) on sequencing data was carried out. Raw data and alignment QC were performed using QC3 (ref. 65), and expression analyses were carried out using MultiRankSeq^66^. All data passed QC. Raw data were aligned with TopHat 2 against mouse MM10 genome, and read count data were obtained using HTSeq, DESeq^67^, edgeR^68^ and baySeq^69^. All software are available on request. False discovery rate<0.05 was used to correct for multiple testing. The resulting ranked canonical pathways (as shown in Fig. 3) were subjected to gene associated feature enrichment analysis using Ingenuity Pathway Analysis (http://www.ingenuity.com).

### Euglycemic-hyperinsulinemic clamps

Insulin sensitivity assessments were conducted on DIO, GB-IL and RYGB mice (*n* of 4–5) at 4 weeks post-operative by the Vanderbilt MMPC. Mice were provided diets and water *ad libitum* and housed at standard room temperature of (23 °C) on a 12 h light cycle. One week before the clamp procedure, catheters were surgically placed in the carotid artery and jugular vein for sampling and infusions, respectively. Mice were withheld food for 5 h prior to the clamp procedure. Erythrocytes were replaced to prevent a decline in haematocrit that occurs with repeated blood sampling. A primed (1.5 μCi) continuous (0.075 μCi min^−1^) [3-^3^H]glucose infusion was started at −20 min. The clamp was initiated at 0 min with a continuous insulin infusion (2.5 mU kg^−1^ min^−1^) that was maintained for 145 min. Arterial glucose was measured at 10 min intervals to provide feedback to adjust the glucose infusion rate as needed to clamp glucose concentration.

### Lipid analyses

Sera were collected from untreated lean, DIO, biliary diverted and RYGB mice after 4 h fasting and stored at −80 °C. Faeces were collected over a 24-h period. Faeces (100 mg) was extracted 3:1 with chloroform:methanol, and the resulting lipid extract evaporated to dryness and weighed. FFAs were analysed by the NEFAs analysis kit (Wako Life Sciences, Richmond VA). Serum TG and cholesterol were analysed using Infinity reagents (Thermo Scientific, Middletown, VA).

### Immunoblot analyses

The liver and distal ileum was collected at 8 weeks post surgery, homogenized in lysis buffer and pelleted. Protein concentrations of the supernatant were analysed, and equivalent amounts of protein were loaded onto a polyacrylamide gel. Primary antibodies raised against TGR5 (ab72608; 1:1,000), CYP7A1 (ab65596, 1:1,000) were purchased from Abcam (Cambridge, MA). Goat anti-FXR (sc-1204; 1:1,000), rabbit anti-OATPβ (sc-135099; 1:500), was from Santa Cruz Biotechnology (Santa Cruz, CA). Sheep anti-FGF15 (1:1,000) was purchased from R&D Systems (Minneapolis, MN). Rabbit anti-SHP2 was a gift from Dr Gen-Sheng Feng, the University of California San Diego, CA. The primary antibodies were diluted in Li-Cor blocking buffer and incubated at room temperature for 1 h. Goat anti-rabbit, donkey anti-goat, goat anti-mouse or donkey anti-sheep fluorescently labelled secondary antibodies (Li-Cor Inc., Lincoln, NE) were diluted 1:10,000 and incubated in blocking buffer at room temperature for 1 h with shaking. After three rinses with PBS solution, the membrane was scanned with the Odyssey Infrared Imaging System (Li-Cor Inc.).

### Caecal content sampling and microbiota analysis

Caecal content samples were collected 8 weeks after surgery and stored at −80 °C when mice were killed. The DNA extractions, amplification, library prep and sequencing were done by the Gut Microbiome Core, at the University of California at Davis. The 16S universal Eubacteria primers (PCR primers 515/806) were used to amplify the V4 variable region. A single-step 30 cycle PCR using HotStarTaq Plus Master Mix Kit (Qiagen, Valencia, CA) was used under the following conditions: 94 °C for 3 min, followed by 28 cycles of 94 °C for 30 s; 53 °C for 40 s and 72 °C for 1 min; after which a final elongation step at 72 °C for 5 min was performed. Following PCR, all amplicon products from different samples were mixed in equal concentrations and purified using Agencourt Ampure beads (Agencourt Bioscience Corporation, MA, USA). Microbial sequencing was analysed by bacterial tag encoded FLX amplicon pyrosequencing (bTEFAP) using a Roche 454 pyrosequencer and titanium reagents and 3–5 K nominal sequences per sample of high quality extracted DNA.

Sequences were depleted of barcodes and primers then short sequences <200 bp are removed, sequences with >1 ambiguous base calls removed and sequences with homopolymer runs exceeding 6 bp removed using the statistical software package Quantitative Insights Into Microbial Ecology (QIIME). A total number of 491,009 sequences passed a quality filter with a minimum score of 25 and an average length of 460 bp. Operational taxonomic units were defined after removal of chimeric and singleton sequences, clustering at 3% divergence (97% similarity)^70^. Operational taxonomic units were then taxonomically classified using BLASTn against a curated GreenGenes database.

For RT–PCR analyses, DNA was extracted from 50 mg of caecal contents on a QIAsymphony (Qiagen 9001297) following Qiagen guidelines using the Complex_200_V6_DSP protocol and the FIX labware. The optional lysozyme pretreatment was performed for each sample. For each faecal sample, 280 μl of Buffer ATL (Qiagen 939011) and 20 μl 10 × Lysozyme (Sigma-Aldrich, St Louise, MO) was added. The samples were vortexed quickly then incubated at 37 °C for 30 min. After incubation, samples were spun briefly to remove condensation from the caps and 300 μl was transferred to 2.0 ml tube (Sarstedt 72.694). Default internal controls were used during the extraction, provided in QIAsymphony DSP Virus/Pathogen Mini Kit (Qiagen 937036). Samples were then eluted to 110 μl AVE. The absolute abundance of *C. minuta* was determined by 16S rRNA gene (Genbank Accession number AB490809) RT–PCR using genomic *C. minuta* (YIT 12065; DSMZ No. 22607, Leibniz-Institut DSMZ-Deutsche Sammlung von Mikroorganismen und Zellkulturen GmbH, Braunschweig, Germany) and primers in Supplementary Table 6.

### Real-time PCR

The liver and ileal samples obtained at 8 weeks post surgery and were subjected to total RNA extraction using Trizol reagent (Invitrogen). After RNA quantification, 2 μg of total RNA was digested with RNase-free DNase I (Roche Diagnostics, Indianapolis, IN), followed by reverse transcription using the High Capacity cDNA Reverse Transcription Kit (Applied Biosystems, Foster City, CA). Relative levels of amplificants were determined using SYBR Green qPCR Supermix (Invitrogen) on a Fast 7900HT Real-Time PCR System (Applied Biosystems) with Integrated DNA Technologies (Coralville, IA) primer pairs in Supplementary Table 6. Standard curves for each target were generated and the efficiency of quantitative RT–PCR for each gene was calculated. All data were normalized to the house-keeping gene, 18S, and a comparative threshold cycle (Ct) method, 2^−ΔΔ^ Ct, was used to compare the relative expression levels.

### Statistics

Sample size for this study was based on a prior sample size calculation guided by the outcome variable of weight loss achieved 4 weeks after RYGB was previously reported.^24^ This calculation indicated that, if using a repeated-measures model, we needed a minimum of eight mice per treatment group to detect statistical significance between groups. All data are expressed as mean±s.e.m. Unless otherwise indicated, one-way analysis of variance with Dunn's post-test was used to compare three or more groups while Student's *t*-test (unpaired, two tailed) was used for binary comparisons. All statistical analyses were performed using Prism version 5.0d software (GraphPad, La Jolla, CA). Differences in microbiota richness were tested by analysis of variance. The threshold of statistical significance was set at *P*<0.05.

## Additional information

**Accession codes**: RNA-seq data have been deposited in NCBI Gene Expression Omnibus (GEO) under accession code GSE68812. Microbiome sequencing data have been deposited in Dryad under the identifier doi:10.5061/dryad.81h12.

**How to cite this article:** Flynn, C. R. *et al*. Bile diversion to the distal small intestine has comparable metabolic benefits to bariatric surgery. *Nat. Commun.* 6:7715 doi: 10.1038/ncomms8715 (2015).

## Supplementary Material

Supplementary Information Supplementary Figures 1-9 and Supplementary Tables 1-6
