# Supplementary material for: Bile diversion to the distal small intestine has comparable metabolic benefits to bariatric surgery
Source: Nat Commun. 2015 Jul 21;6:7715. doi: 10.1038/ncomms8715 (PMC4518285; doi:10.1038/ncomms8715)
Supplement: Supplementary Information — Supplementary Figures 1-9 and Supplementary Tables 1-6 [file ncomms8715-s1.pdf]

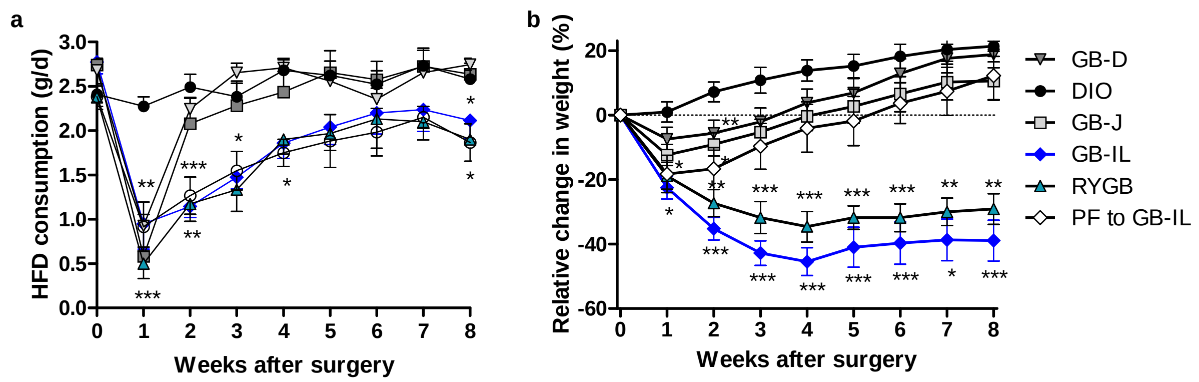

Supplemental Figure 1. (**a**) HFD consumption and (**b**) relative changes in body weight of DIO, GB-D, GB-J, GB-IL, RYGB and pair-fed to GB-IL mice up to eight weeks post-op. * $P < 0.05$, **$P<0.01$, ***$P<0.001$ versus DIO controls. Values shown are means ± SEM (one-way ANOVA with Dunn's post-test). N=15 DIO, 14 GB-D, 14 GB-J, 15 GB-IL, 7 RYGB, 6 PF to GB-IL.

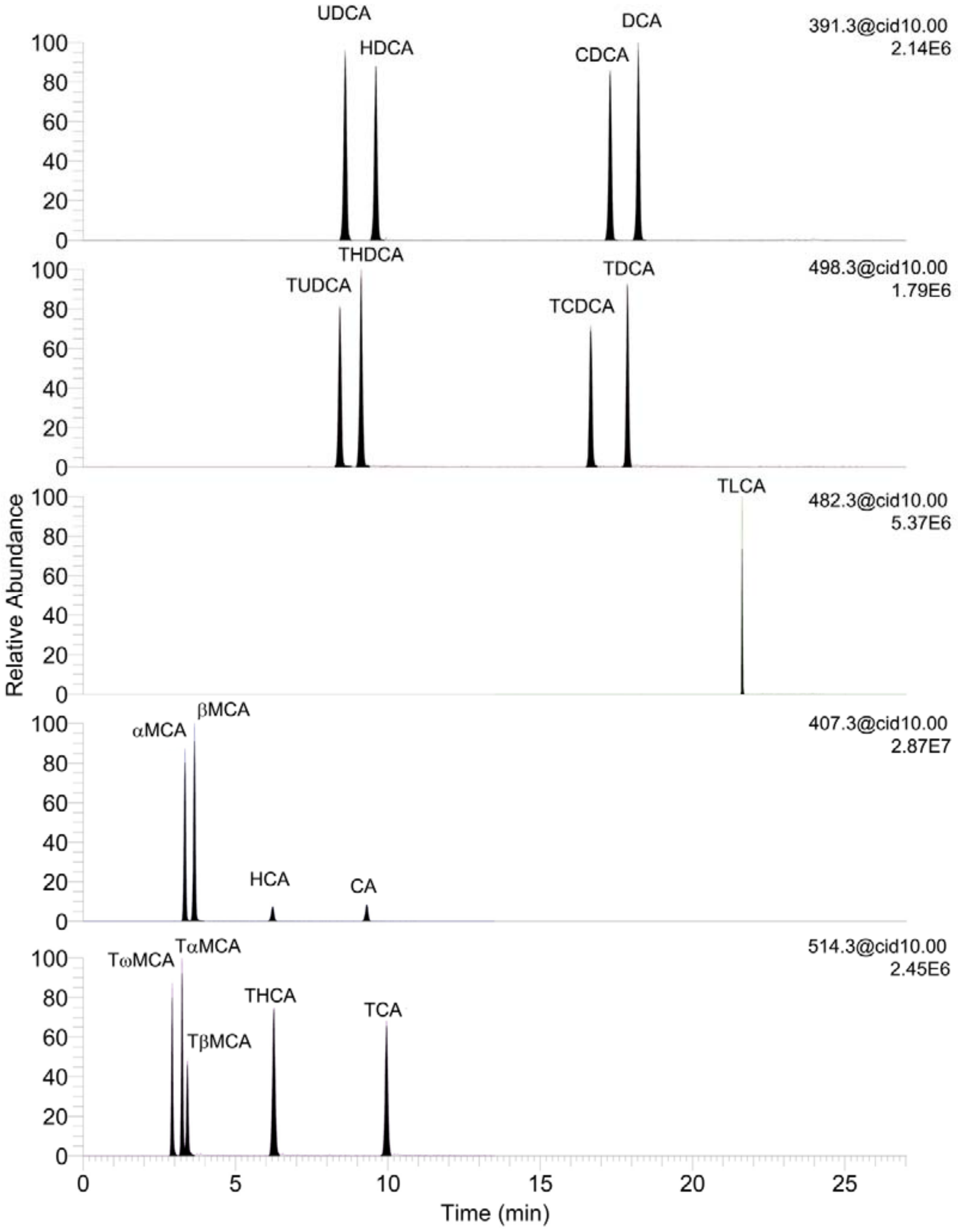

Supplemental Figure 2. Extracted ion chromatograms of serum bile acids resolved by high-performance liquid chromatographic ESI-MS/MS. Individual samples spikes with deuterated bile acid standards were resolved over a 28 min gradient. Eluting bile acid species were subjected to mass analysis and area under the curve (AUC) determinations were made.

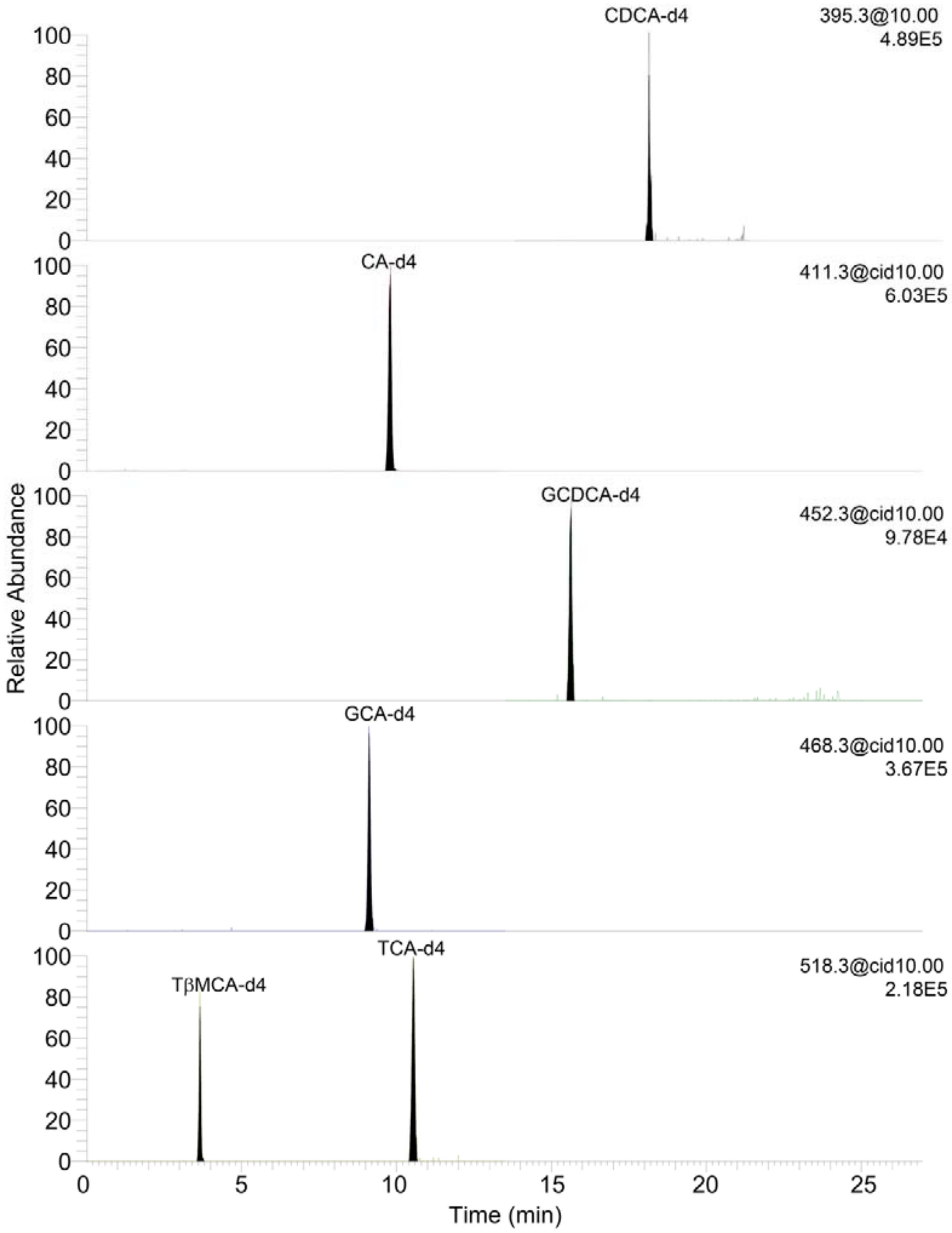

Supplemental Figure 3. Extracted ion chromatograms of deuterated bile acids standards used as internal standards in the quantitation analysis. Individual samples spiked with deuterated bile acid standards were resolved over a 28 min gradient and used to quantify bile acid species as described in Materials and Methods.

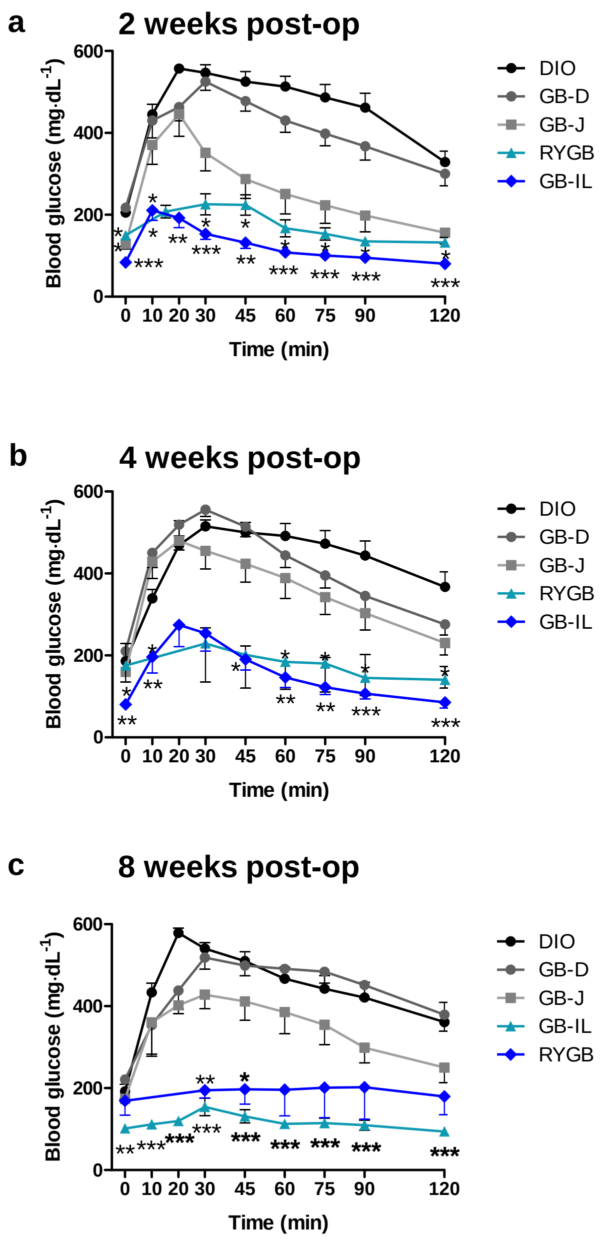

Supplemental Figure 4. Glucose excursion curves in DIO WT, bile diversion and RYGB mice after Intraperitoneal glucose tolerance tests (IPGTT) at A) 2 weeks post-op, B) 4 weeks post-op, and c) 8 weeks post-op. Data are shown as mean ± SEM. **P<0.05*, ***P*<0.01, ****P*< 0.001 versus DIO by one-way ANOVA with post-test.

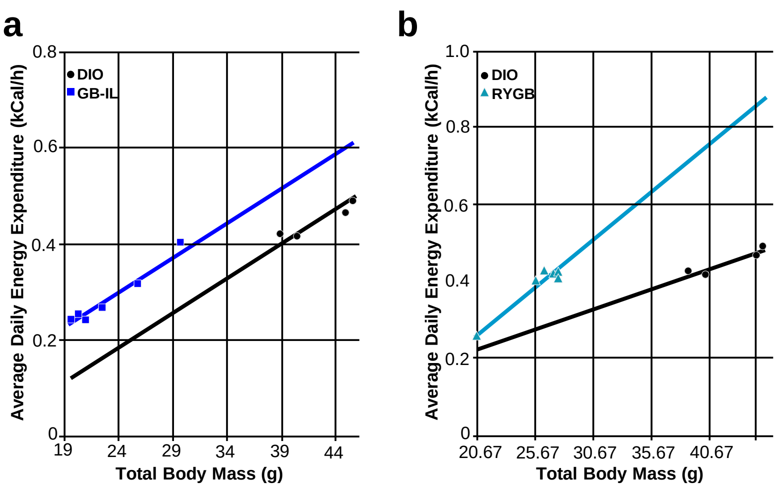

Supplemental Figure 5. Regression lines for ANCOVA predicted energy expenditure adjusted for total body mass (TBM) when comparing GB-IL (N=6) versus DIO (N=4) (**a**) and RYGB (N=6) versus DIO (N=4) (**b**). Energy expenditure using indirect calorimetry was measured 4 weeks after surgical procedures on diet-induced obese (DIO) mice and analyzed by analysis of covariance using surgical procedure as main effects and testing for interactions as described in the Materials and Methods and on the Mouse Metabolic Phenotyping Center website (http://www.mmpc.org). GB-IL mice compared to DIO mice displayed parallel linear regression lines suggestive of an increased energy expenditure that was independent of a TBM interaction (**a**). In contrast, ANCOVA-adjusted comparisons of RYGB to DIO controlling for TBM indicated an increased energy expenditure in RYGB mice that was dependent on a TBM interaction (**b**).

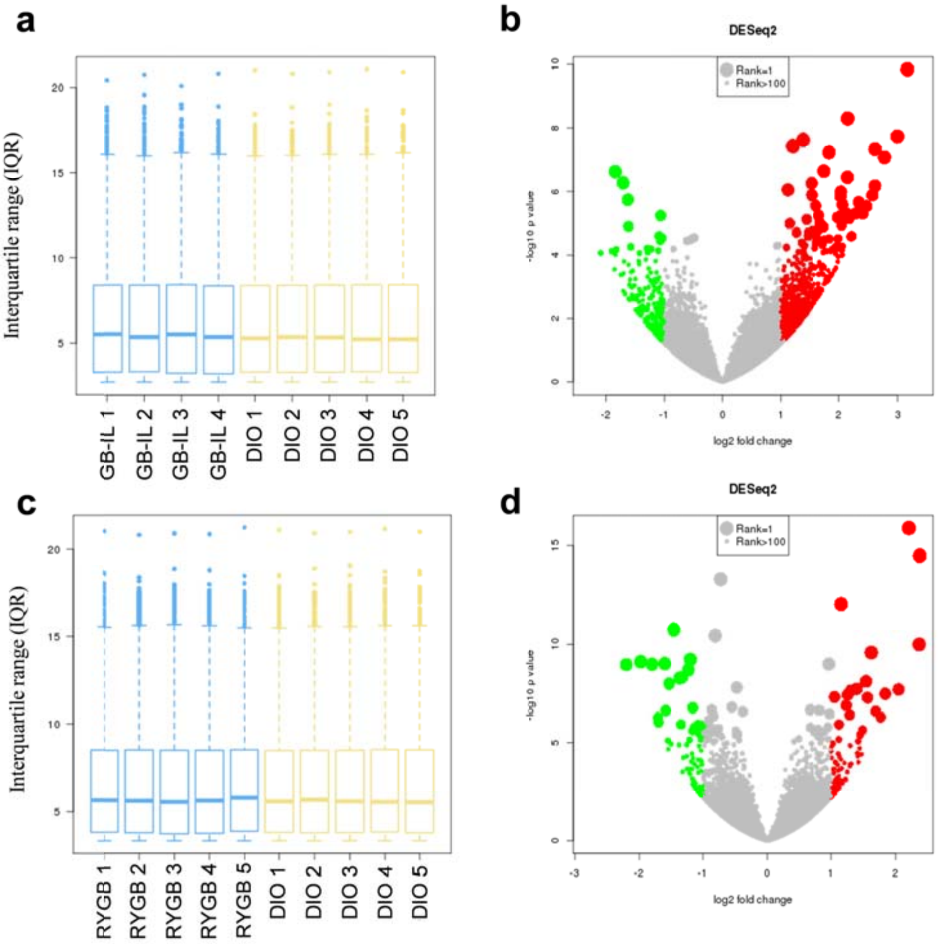

Supplementary Figure 6. RNA-seq analysis (all transcripts represented, filtered to remove zero values; 25,212 transcripts) from GB-IL and DIO mouse livers (**a**,**b**) or RYGB and DIO mouse livers (**c**,**d**). (**a**, **c**) Box plot depicting the difference among RNA-seq datasets after normalization by Robust Microarray Averaging (RMA). Boxes show the 25th and 75th percentiles in the distribution of log-transformed (log base 2) intensities. The median is the horizontal bar in the middle of the box. The whiskers (dotted lines extending from the boxes) illustrate the maximum value or 1.5 times the interquartile range of data (IQR), whichever is smaller. The circles display any points beyond these whiskers. (**b**,**d**). Volcano plots of log2 fold-change (x-axis) versus −log10 FDR-corrected p-value (y-axis, representing the probability that the gene is differentially expressed) in RNA-seq data of mouse livers from GB-IL vs DIO (**b**) and RYGB vs DIO mice (**d**).

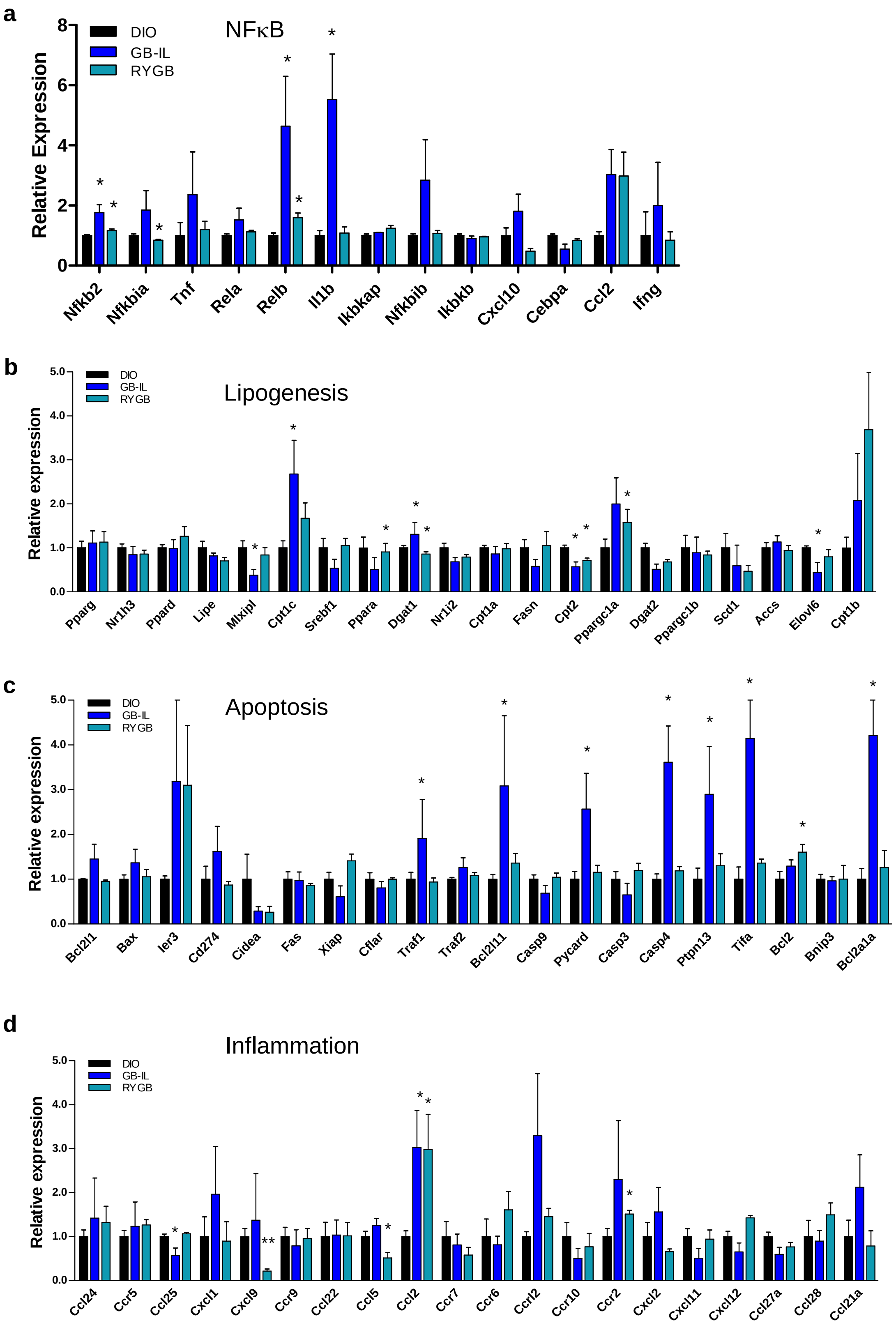
a
NFκB
DIO
GB-IL
RYGB
Relative Expression
8
6
4
2
0
Nfkb2
Nfkbia
Tnf
Rela
Relb
Il1b
Ikbkap
Nfkbib
Ikbkb
Cxcl10
Cebpa
Ccl2
Ifng
b
Lipogenesis
DIO
GB-IL
RYGB
Relative expression
5.0
4.0
3.0
2.0
1.0
0.0
Pparg
Nr1h3
Ppard
Lipe
Mlxipl
Cpt1c
Srebf1
Ppara
Dgat1
Nr1i2
Cpt1a
Fasn
Cpt2
Ppargc1a
Dgat2
Ppargc1b
Scd1
Accs
Elovl6
Cpt1b
c
Apoptosis
DIO
GB-IL
RYGB
Relative expression
5.0
4.0
3.0
2.0
1.0
0.0
Bcl2l1
Bax
Ier3
Cd274
Cidea
Fas
Xiap
Cflar
Traf1
Traf2
Bcl2l11
Casp9
Pycard
Casp3
Casp4
Ptpn13
Tifa
Bcl2
Bnip3
Bcl2a1a
d
Inflammation
DIO
GB-IL
RYGB
Relative expression
5.0
4.0
3.0
2.0
1.0
0.0
Ccl24
Ccr5
Ccl25
Cxcl1
Cxcl9
Ccr9
Ccl22
Ccl5
Ccl2
Ccr7
Ccr6
Ccrl2
Ccr10
Ccr2
Cxcl2
Cxcl11
Cxcl12
Ccl27a
Ccl28
Ccl21a

Supplementary Figure 7. Bile diversion to the ileum (GB-IL) and RYGB have differential effects on hepatic gene expression. RNA-seq analysis of (**a**) NFκB-related genes, (**b**) lipogenesis genes, (**c**) apoptosis genes and (**d**) inflammatory genes in the livers of DIO, GB-IL and RYGB mice as assigned to canonical pathways curated by Ingenuity Pathway Analysis. Expression was normalized as described in Supplementary Materials and Methods. N= 4-5 mice per group. Data are presented as mean ± SEM, unpaired two-tailed Student's *t*-test. **P*<0.05, **P*<0.01 compared with DIO controls.

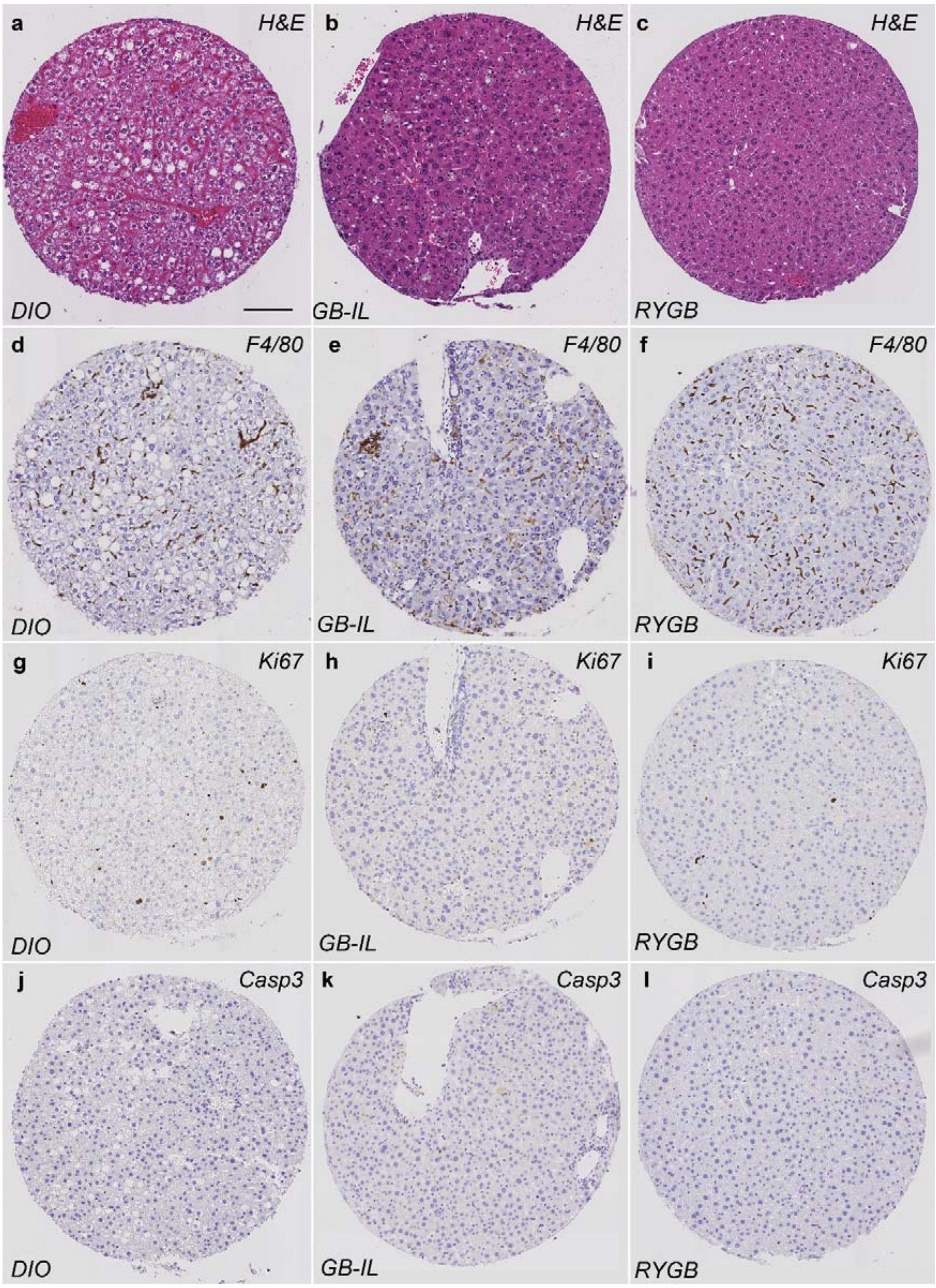

Supplemental Figure 8. Representative micrographs of DIO, GB-IL and RYGB mouse livers at 4 weeks post-operative stained for hematoxylin and eosin (**a-c**) and F4/80 (**d-f**), Ki-67 (**g-i**) and caspase 3 (**j-l**) revealed by DAB reactivity and counter-stained for eosin. Mag bar = 100 μm.

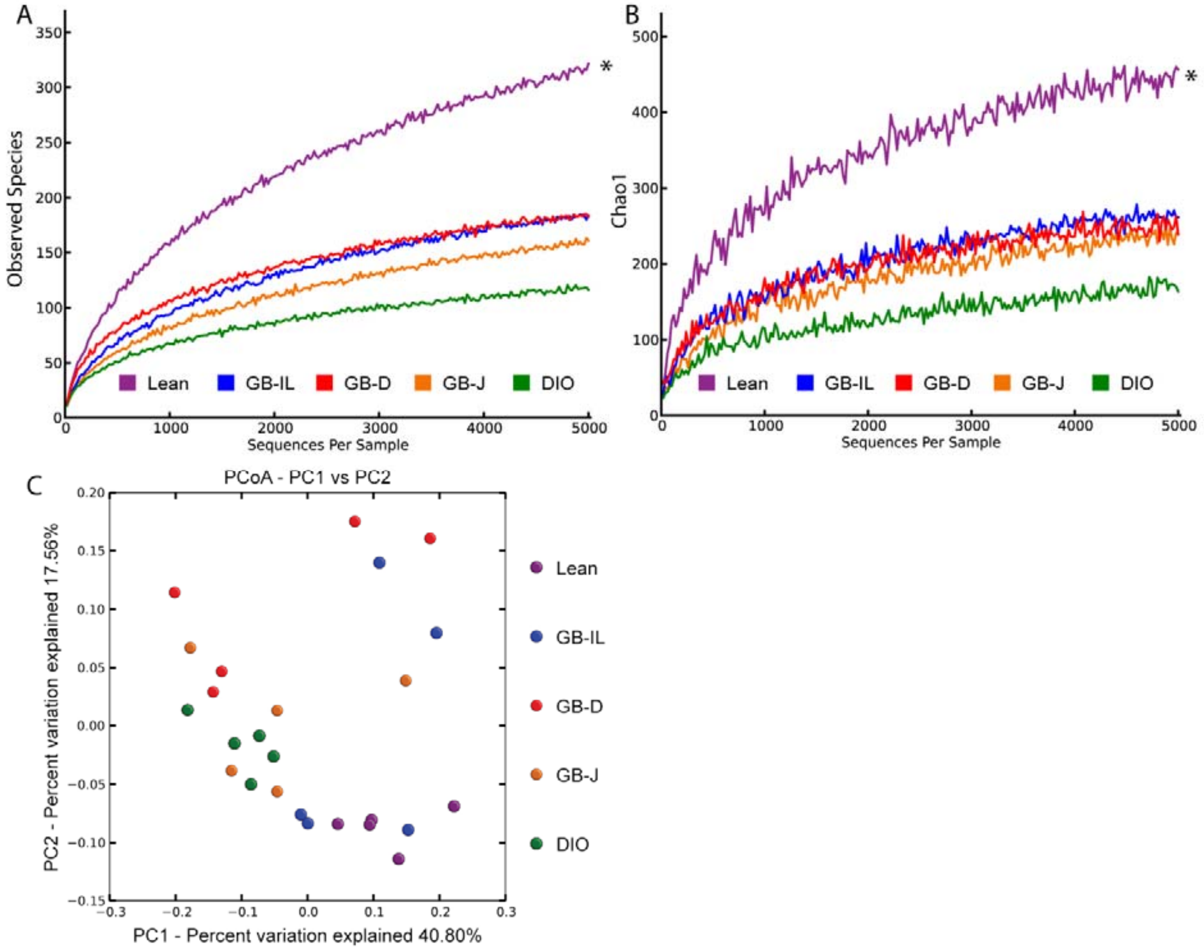

Supplemental Figure 9. High fat diet decreases gut microbial diversity. Cecal contents were obtained 8 weeks after surgery. Microbial diversity was analyzed by bacterial tag encoded FLX amplicon pyrosequencing (bTEFAP) using a Roche 454 pyrosequencer and titanium reagents, and 3-5k nominal sequences per sample of high quality extracted DNA (**a**) Rarefaction curves (OUT at sequences dissimilarity cutoff < 3%). Rarefaction curves were calculated for OTUs and indicated that the analysis neared plateau but could benefit from additional sequencing. (**b**) Chao1 estimates of gut microbial diversity. *P < 0.05 (**c**) Principal component analysis indicating each sample microbiota variance relative to each other. N= 5 per group.

Supplemental Table 1. Analytical settings for detected bile acids.

Species	m/z	R.T.	Internal Standard
TωMCA	514.3	2.72	TβMCA-d4
ωMCA	407.3	2.80	CA-d4
TαMCA	514.3	3.01	TβMCA-d4
αMCA	407.3	3.17	CA-d4
TβMCA	514.3	3.17	TβMCA-d4
βMCA	407.3	3.49	CA-d4
THCA	514.3	5.80	GCA-d4
HCA	407.3	6.22	CA-d4
TUDCA	498.3	8.06	GCDCA-d4
UDCA	391.3	8.37	CDCA-d4
THDCA	498.3	8.70	GCDCA-d4
CA	407.3	9.00	CA-d4
HDCA	391.3	9.36	CDCA-d4
TCA	514.3	9.50	TCA-d4
TCDCA	498.3	16.19	TCA-d4
CDCA	391.3	17.00	CDCA-d4
TDCA	498.3	17.48	TCA-d4
DCA	391.3	17.94	CDCA-d4
TLCA	482.3	21.59	CDCA-d4
**GCDCA-d4**	452.3	14.50	
**CDCA-d4**	395.3	17.00	
**TCA-d4**	518.3	9.50	
**CA-d4**	411.3	9.00	
**TβMCA-d4**	518.3	3.17	
**GCA-d4**	468.3	8.14	

Bold: deuterated internal standards

Supplemental Table 2. Mean ± SEM bile acid species levels in bile diverted and RYGB mouse serum.

**BA Species**	**Obese**	**GB-D**	**GB-J**	**GB-IL**	**RYGB**
TωMCA	291.1 ± 80.2	291.8 ± 157	376.9 ± 81.2	1609.6 ± 437.4	185.9 ± 74.8
TβMCA	1145.3 ± 273.6	662 ± 234.1	692.3 ± 257.9	9741.9 ± 2040.2	947.3 ± 515.9
TαMCA	179.7 ± 50.6	131.3 ± 77.6	128.3 ± 50.9	110.9 ± 34	89.3 ± 42.1
CA	191.6 ± 51.2	544 ± 356.9	199.4 ± 80.5	76.4 ± 13.5	485.5 ± 427.1
TCDCA	46.1 ± 11.3	18.7 ± 39.2	25.1 ± 9.4	29.9 ± 13	17 ± 2.3
DCA	51.6 ± 8.6	120.8 ± 77.2	24.4 ± 8.6	28.4 ± 14.3	76.5 ± 46.4
TCA	1216.9 ± 369.6	665.6 ± 163.6	740.5 ± 166.6	2202.1 ± 846.6	476.2 ± 245.4
βMCA	15.2 ± 0.6	30.8 ± 7.9	0 ± 0	42.5 ± 5.6	38.5 ± 14.9
TUDCA	42.5 ± 12.3	51 ± 20.2	41.1 ± 10.8	64.5 ± 11.6	0 ± 0
TDCA	11.1 ± 3.2	167.2 ± 77.3	40.2 ± 14.1	21.3 ± 5.4	31.7 ± 10.3
THDCA	10.8 ± 3.8	55.4 ± 11.7	24.7 ± 7.5	32.9 ± 13.4	62.1 ± 24.8
CDCA	5.8 ± 0	64 ± 45.3	22.4 ± 15.8	0 ± 0	38.2 ± 27

n = 5 mice per group

Supplemental Table 3. Basic mean and two-way ANOVA contrasts adjusted for total body mass (TBM) and fat-free mass (FFM).

			Basic Stats				Model Overall					
Covariate	Phase	Variable	DIO	GBIL	DIO	RYGB	DIO	GBIL	p-value	DIO	RYGB	p value
TBM	Light	Avg_EE	0.42 (0.031)	0.28 (0.037)	0.42 (0.031)	0.36 (0.052)	0.3 (0.013)	0.36 (0.009)	0.016	0.32 (0.03)	0.5 (0.019)	0.003
FFM	Light	Avg_EE	0.42 (0.031)	0.28 (0.037)	0.42 (0.031)	0.36 (0.052)	0.36 (0.012)	0.32 (0.009)	0.050	0.37 (0.017)	0.4 (0.013)	0.241
TBM	Light	Tot_EE	4.32 (0.321)	2.9 (0.391)	4.32 (0.321)	3.75 (0.547)	3.07 (0.135)	3.73 (0.093)	0.019	3.31 (0.32)	5.13 (0.203)	0.003
FFM	Light	Tot_EE	4.32 (0.321)	2.9 (0.391)	4.32 (0.321)	3.75 (0.547)	3.74 (0.122)	3.28 (0.09)	0.040	3.78 (0.169)	4.11 (0.129)	0.227
TBM	Light	Avg_VO2	1.45 (0.108)	0.98 (0.134)	1.45 (0.108)	1.26 (0.18)	1.03 (0.047)	1.26 (0.033)	0.018	0.93 (0.099)	1.61 (0.068)	0.004
FFM	Light	Avg_VO2	1.45 (0.108)	0.98 (0.134)	1.45 (0.108)	1.26 (0.18)	1.26 (0.044)	1.1 (0.033)	0.055	1.28 (0.058)	1.38 (0.044)	0.269
TBM	Light	Avg_VCO2	1.13 (0.087)	0.79 (0.087)	1.13 (0.087)	0.99 (0.145)	0.83 (0.037)	0.99 (0.025)	0.035	0.85 (0.079)	1.36 (0.05)	0.002
FFM	Light	Avg_VCO2	1.13 (0.087)	0.79 (0.087)	1.13 (0.087)	0.99 (0.145)	0.99 (0.032)	0.88 (0.023)	0.051	0.98 (0.045)	1.09 (0.034)	0.143
TBM	Light	Avg_RQ	0.77 (0.007)	0.81 (0.028)	0.77 (0.007)	0.79 (0.008)	0.81 (0.03)	0.78 (0.021)	0.639	0.76 (0.011)	0.79 (0.008)	0.107
FFM	Light	Avg_RQ	0.77 (0.007)	0.81 (0.028)	0.77 (0.007)	0.79 (0.008)	0.79 (0.018)	0.8 (0.014)	0.747	0.77 (0.005)	0.79 (0.004)	0.031
TBM	Dark	Avg_EE	0.48 (0.032)	0.29 (0.078)	0.48 (0.032)	0.41 (0.078)	0.36 (0.044)	0.46 (0.021)	0.081	0.38 (0.044)	0.61 (0.028)	0.004
FFM	Dark	Avg_EE	0.48 (0.032)	0.29 (0.078)	0.48 (0.032)	0.41 (0.078)	0.38 (0.022)	0.36 (0.016)	0.599	0.41 (0.021)	0.46 (0.016)	0.108
TBM	Dark	Tot_EE	5.76 (0.383)	3.51 (0.941)	5.76 (0.383)	4.95 (0.93)	4.28 (0.534)	5.52 (0.25)	0.081	4.5 (0.524)	7.28 (0.332)	0.004
FFM	Dark	Tot_EE	5.76 (0.383)	3.51 (0.941)	5.76 (0.383)	4.95 (0.93)	4.54 (0.261)	4.33 (0.193)	0.599	4.87 (0.253)	5.54 (0.192)	0.108
TBM	Dark	Avg_VO2	1.66 (0.11)	1.01 (0.274)	1.66 (0.11)	1.42 (0.263)	0.92 (0.121)	1.51 (0.084)	0.020	1.3 (0.154)	2.08 (0.098)	0.005
FFM	Dark	Avg_VO2	1.66 (0.11)	1.01 (0.274)	1.66 (0.11)	1.42 (0.263)	1.31 (0.077)	1.25 (0.057)	0.629	1.41 (0.072)	1.59 (0.055)	0.131
TBM	Dark	Avg_VCO2	1.32 (0.092)	0.8 (0.206)	1.32 (0.092)	1.16 (0.233)	0.75 (0.087)	1.18 (0.06)	0.018	1.02 (0.119)	1.75 (0.076)	0.002
FFM	Dark	Avg_VCO2	1.32 (0.092)	0.8 (0.206)	1.32 (0.092)	1.16 (0.233)	1.04 (0.055)	0.98 (0.04)	0.469	1.09 (0.063)	1.3 (0.048)	0.056
TBM	Dark	Avg_RQ	0.79 (0.01)	0.79 (0.024)	0.79 (0.01)	0.81 (0.027)	0.8 (0.028)	0.78 (0.02)	0.744	0.75 (0.028)	0.84 (0.02)	0.087
FFM	Dark	Avg_RQ	0.79 (0.01)	0.79 (0.024)	0.79 (0.01)	0.81 (0.027)	0.79 (0.017)	0.79 (0.013)	0.860	0.77 (0.014)	0.82 (0.011)	0.059
TBM	Total	Avg_EE	0.45 (0.031)	0.29 (0.057)	0.45 (0.031)	0.39 (0.064)	0.28 (0.02)	0.4 (0.014)	0.010	0.35 (0.033)	0.55 (0.021)	0.002
FFM	Total	Avg_EE	0.45 (0.031)	0.29 (0.057)	0.45 (0.031)	0.39 (0.064)	0.37 (0.015)	0.34 (0.011)	0.221	0.39 (0.018)	0.43 (0.014)	0.137
TBM	Total	Tot_EE	10.08 (0.699)	6.41 (1.325)	10.08 (0.699)	8.7 (1.467)	6.29 (0.48)	8.94 (0.333)	0.011	7.81 (0.763)	12.41 (0.484)	0.002
FFM	Total	Tot_EE	10.08 (0.699)	6.41 (1.325)	10.08 (0.699)	8.7 (1.467)	8.29 (0.346)	7.61 (0.256)	0.234	8.65 (0.403)	9.65 (0.306)	0.131
TBM	Total	Avg_VO2	1.56 (0.108)	0.99 (0.203)	1.56 (0.108)	1.34 (0.219)	0.98 (0.074)	1.38 (0.051)	0.011	1.21 (0.116)	1.9 (0.073)	0.002
FFM	Total	Avg_VO2	1.56 (0.108)	0.99 (0.203)	1.56 (0.108)	1.34 (0.219)	1.28 (0.055)	1.18 (0.041)	0.243	1.34 (0.062)	1.48 (0.047)	0.162
TBM	Total	Avg_VCO2	1.22 (0.089)	0.79 (0.145)	1.22 (0.089)	1.08 (0.187)	0.79 (0.047)	1.08 (0.033)	0.007	0.94 (0.086)	1.55 (0.054)	0.001
FFM	Total	Avg_VCO2	1.22 (0.089)	0.79 (0.145)	1.22 (0.089)	1.08 (0.187)	1.02 (0.036)	0.93 (0.027)	0.150	1.04 (0.051)	1.2 (0.039)	0.069
TBM	Total	Avg_RQ	0.78 (0.009)	0.8 (0.025)	0.78 (0.009)	0.8 (0.015)	0.8 (0.029)	0.78 (0.02)	0.685	0.75 (0.016)	0.82 (0.011)	0.046
FFM	Total	Avg_RQ	0.78 (0.009)	0.8 (0.025)	0.78 (0.009)	0.8 (0.015)	0.79 (0.017)	0.79 (0.013)	0.934	0.77 (0.008)	0.8 (0.006)	0.020

For ANOVA of main effects, cells indicating *$P \leq 0.05$ are shaded pink, cells indicating *$P \leq 0.01$ are shaded red and cells indicating *$P \leq 0.001$ are shaded dark red. (n=4 DIO, 6 GB-IL and 6 RYGB). Indirect calorimetry parameters were compared by a 3-way analysis of variances using surgical procedure (GB-IL or RYGB) as main effects and testing for interactions with covariates being either total body mass (TBM) or fat-free mass (FFM; italics). Least squares means were compared using residual variance as the error term and presented as mean ± SEM. Abbreviations: Avg EE, mean energy expenditure in kcal/hr; Tot_EE, Summed energy expenditure for entire cycle; Avg_VO2, mean volume of O_2 ml/min; Avg_VCO2, mean volume of CO_2 ml/min; Avg_RQ, mean respiratory quotient).

Supplemental Table 4. Weighted Unifrac significance test

Group		*P* value
DIO	Lean	**0.03**
GB-D	GB-IL	**0.05**
	GB-J	0.62
	HFD	0.39
	Lean	**0.04**
GB-J	DIO	0.87
	Lean	**0.00**
GB-IL	GB-J	**0.03**
	DIO	0.06
	Lean	0.06

Supplemental Table 5. OTU table.

Taxon	Lean	GB-IL	GB-D	GB-J	DIO
None;Other;Other;Other;Other;Other	7159	7186	4041	5991	6392
k__Bacteria;Other;Other;Other;Other;Other	2	0	0	1	0
k__Bacteria;p__Actinobacteria;c__Actinobacteria;o__Bifidobacteriales;f__Bifidobacteriaceae;g__Bifidobacterium	25	103	8	54	38
k__Bacteria;p__Actinobacteria;c__Thermoleophilia;o__Gaiellales;f__Gaiellaceae;g__	2	0	2	0	0
k__Bacteria;p__Bacteroidetes;c__Bacteroidia;o__Bacteroidales;Other;Other	4	0	0	0	0
k__Bacteria;p__Bacteroidetes;c__Bacteroidia;o__Bacteroidales;f__;g__	36	0	0	11	0
k__Bacteria;p__Bacteroidetes;c__Bacteroidia;o__Bacteroidales;f__Bacteroidaceae;g__Bacteroides	2329	1338	7	18	5
k__Bacteria;p__Bacteroidetes;c__Bacteroidia;o__Bacteroidales;f__Porphyromonadaceae;g__Parabacteroides	250	404	0	26	0
k__Bacteria;p__Bacteroidetes;c__Bacteroidia;o__Bacteroidales;f__Prevotellaceae;g__Prevotella	276	0	0	1	0
k__Bacteria;p__Bacteroidetes;c__Bacteroidia;o__Bacteroidales;f__Rikenellaceae;g__	2607	80	258	125	27
k__Bacteria;p__Bacteroidetes;c__Bacteroidia;o__Bacteroidales;f__Rikenellaceae;g__AF12	38	1	0	27	2
k__Bacteria;p__Bacteroidetes;c__Bacteroidia;o__Bacteroidales;f__S24-7;g__	10038	117	85	87	5
k__Bacteria;p__Bacteroidetes;c__Bacteroidia;o__Bacteroidales;f__[Odoribacteraceae];g__Odoribacter	64	1	6	44	0
k__Bacteria;p__Deferribacteres;c__Deferribacteres;o__Deferribacterales;f__Deferribacteraceae;g__Mucispirillum	14	3	1	24	0
k__Bacteria;p__Firmicutes;c__Bacilli;o__Bacillales;f__Staphylococcaceae;g__Staphylococcus	0	7	0	0	0
k__Bacteria;p__Firmicutes;c__Bacilli;o__Lactobacillales;f__Enterococcaceae;g__Enterococcus	185	2431	301	293	364
k__Bacteria;p__Firmicutes;c__Bacilli;o__Lactobacillales;f__Lactobacillaceae;g__	4002	2366	2819	955	1577
k__Bacteria;p__Firmicutes;c__Bacilli;o__Lactobacillales;f__Lactobacillaceae;g__Lactobacillus	29938	31835	4571	32303	28501
k__Bacteria;p__Firmicutes;c__Bacilli;o__Lactobacillales;f__Streptococcaceae;Other	4	75	42	6	11
k__Bacteria;p__Firmicutes;c__Bacilli;o__Lactobacillales;f__Streptococcaceae;g__Streptococcus	0	2	2	2	0
k__Bacteria;p__Firmicutes;c__Bacilli;o__Turicibacterales;f__Turicibacteraceae;g__Turicibacter	0	3	50	22	440
k__Bacteria;p__Firmicutes;c__Clostridia;Other;Other;Other	20	6	25	10	0
k__Bacteria;p__Firmicutes;c__Clostridia;o__;f__;g__	54	18	5	9	2
k__Bacteria;p__Firmicutes;c__Clostridia;o__Clostridiales;Other;Other	7869	888	1	3	21
k__Bacteria;p__Firmicutes;c__Clostridia;o__Clostridiales;f__;g__	37	0	7	5	0
k__Bacteria;p__Firmicutes;c__Clostridia;o__Clostridiales;f__Clostridiaceae;g__	96	32	39	21	12
k__Bacteria;p__Firmicutes;c__Clostridia;o__Clostridiales;f__Clostridiaceae;g__Clostridium	0	1548	3172	1019	629
k__Bacteria;p__Firmicutes;c__Clostridia;o__Clostridiales;f__Dehalobacteriaceae;g__Dehalobacterium	48	3	2	1	1
k__Bacteria;p__Firmicutes;c__Clostridia;o__Clostridiales;f__Lachnospiraceae;Other	354	578	3144	990	221
k__Bacteria;p__Firmicutes;c__Clostridia;o__Clostridiales;f__Lachnospiraceae;g__	1639	616	3015	1391	235
k__Bacteria;p__Firmicutes;c__Clostridia;o__Clostridiales;f__Lachnospiraceae;g__Anaerostipes	36	0	0	0	0
k__Bacteria;p__Firmicutes;c__Clostridia;o__Clostridiales;f__Lachnospiraceae;g__[Ruminococcus]	272	128	1022	13	54
k__Bacteria;p__Firmicutes;c__Clostridia;o__Clostridiales;f__Peptococcaceae;g__	3	0	1	0	0
k__Bacteria;p__Firmicutes;c__Clostridia;o__Clostridiales;f__Peptostreptococcaceae;g__	552	4394	11249	1433	2100
k__Bacteria;p__Firmicutes;c__Clostridia;o__Clostridiales;f__Ruminococcaceae;Other	271	41	42	38	17
k__Bacteria;p__Firmicutes;c__Clostridia;o__Clostridiales;f__Ruminococcaceae;g__	1602	12	26	16	143
k__Bacteria;p__Firmicutes;c__Clostridia;o__Clostridiales;f__Ruminococcaceae;g__Anaerotruncus	7	25	5	0	0
k__Bacteria;p__Firmicutes;c__Clostridia;o__Clostridiales;f__Ruminococcaceae;g__Oscillospira	812	89	62	105	18
k__Bacteria;p__Firmicutes;c__Clostridia;o__Clostridiales;f__Ruminococcaceae;g__Ruminococcus	132	6	13	9	0
k__Bacteria;p__Firmicutes;c__Clostridia;o__Coriobacteriales;f__;g__	5	0	0	0	0
k__Bacteria;p__Firmicutes;c__Clostridia;o__Coriobacteriales;f__Coriobacteriaceae;g__	45	0	0	0	0
k__Bacteria;p__Firmicutes;c__Clostridia;o__Coriobacteriales;f__Coriobacteriaceae;g__Adlercreutzia	1475	198	1895	552	362
k__Bacteria;p__Firmicutes;c__Erysipelotrichi;o__Erysipelotrichales;f__Erysipelotrichaceae;g__	105	2	4	2	4
k__Bacteria;p__Firmicutes;c__Erysipelotrichi;o__Erysipelotrichales;f__Erysipelotrichaceae;g__Allobaculum	7075	55	16044	20682	12585
k__Bacteria;p__Firmicutes;c__Erysipelotrichi;o__Erysipelotrichales;f__[Coprobacillaceae];g__	0	4	0	0	0
k__Bacteria;p__Firmicutes;c__Erysipelotrichi;o__Erysipelotrichales;f__[Coprobacillaceae];g__Coprobacillus	156	0	0	0	0
k__Bacteria;p__Proteobacteria;c__Alphaproteobacteria;o__;f__;g__	6	0	0	0	0
k__Bacteria;p__Proteobacteria;c__Alphaproteobacteria;o__RF32;f__;g__	8	0	0	0	0
k__Bacteria;p__Proteobacteria;c__Betaproteobacteria;o__Burkholderiales;f__Alcaligenaceae;g__Sutterella	258	10	0	0	0
k__Bacteria;p__Proteobacteria;c__Betaproteobacteria;o__Burkholderiales;f__Burkholderiaceae;g__	2	0	0	0	0
k__Bacteria;p__Proteobacteria;c__Deltaproteobacteria;o__Desulfovibrionales;f__Desulfovibrionaceae;g__Bilophila	0	0	0	1	1
k__Bacteria;p__Proteobacteria;c__Deltaproteobacteria;o__Desulfovibrionales;f__Desulfovibrionaceae;g__Desulfovibrio	155	25	219	89	31
k__Bacteria;p__Proteobacteria;c__Epsilonproteobacteria;o__Campylobacterales;f__Helicobacteraceae;Other	38	2	0	0	0
k__Bacteria;p__Proteobacteria;c__Epsilonproteobacteria;o__Campylobacterales;f__Helicobacteraceae;g__Helicobacter	377	1143	0	23	2
k__Bacteria;p__Proteobacteria;c__Gammaproteobacteria;o__Enterobacteriales;f__Enterobacteriaceae;Other	0	2611	26	233	0
k__Bacteria;p__Proteobacteria;c__Gammaproteobacteria;o__Enterobacteriales;f__Enterobacteriaceae;g__Erwinia	0	0	0	2	0
k__Bacteria;p__Proteobacteria;c__Gammaproteobacteria;o__Enterobacteriales;f__Enterobacteriaceae;g__Escherichia	0	1784	46	107	0
k__Bacteria;p__Proteobacteria;c__Gammaproteobacteria;o__Enterobacteriales;f__Enterobacteriaceae;g__Klebsiella	0	5507	0	0	0
k__Bacteria;p__Proteobacteria;c__Gammaproteobacteria;o__Enterobacteriales;f__Enterobacteriaceae;g__Trabulsiella	0	113	1	0	0
k__Bacteria;p__Spirochaetes;c__[Brachyspirae];o__[Brachyspirales];f__Brachyspiraceae;g__Brachyspira	5	0	0	0	0
k__Bacteria;p__TM7;c__TM7-3;o__CW040;f__F16;g__	1333	0	726	851	2108
k__Bacteria;p__Verrucomicrobia;c__Verrucomicrobiae;o__Verrucomicrobiales;f__Verrucomicrobiaceae;g__Akkermansia	0	32	0	19	0

Supplemental Table 6. Oligonucleotides used for quantitative RT-PCR.

Target	Forward Primer	Reverse Primer
BACS	ACC CTG GAT CAG CTC CTG GAT	GTT CTC AGC TAG CAG CTT GG
BAT	GGA AAC CTG TTA GTT CTC AGG C	GTG GAC CCC CAT ATA GTC TCC
BSEP	CTG CCA AGG ATG CTA ATG CA	CGA TGG CTA CCC TTT GCT TCT
IBAT	ACC ACT TGC TCC ACA CTG CTT	CGT TCC TGA GTC AAC CCA CAT
MRP3	TCC CAC TTT TCG GAG ACA GTA AC	ACT GAG GAC CTT GAA GTC TTG GA
NTCP	ATG ACC ACC TGC TCC AGC TT	GCC TTT GTA GGG CAC CTT GT
OATP1	CAG TCT TAC GAG TGT GCT CCA GAT	ATG AGG AAT ACT GCC TCT GAA GTG
OSTα	TGT TCC AGG TGC TTG TCA TCC	CCA CTG TTA GCC AAG ATG GAG AA
OSTβ	GAT GCG GCT CCT TGG AAT TA	GGA GGA ACA TGC TTG TCA TGA C
IBABP	CAG GAG ACG TGA TTG AAA GGG	GCC CCC AGA GTA AGA CTG GG
A. muciniphila	CAG CAC GTG AAG GTG GGG AC	CCT TGC GGT TGG CTT CAG AT
C. minuta	TTC GGG AGG AAC TGT GGG TAT	GGT TGC TCA CGC GTT ACT CA
